# Anticoagulant-related bleeding as a sign of underlying tumoural lesions in patients with atrial fibrillation: a nationwide cohort study

**DOI:** 10.1093/ehjopen/oeae081

**Published:** 2024-09-24

**Authors:** Kristiaan Proesmans, Maxim Grymonprez, Sylvie Rottey, Lies Lahousse

**Affiliations:** Faculty of Pharmaceutical Sciences, Department of Bio-analysis, Pharmaceutical Care Unit, Ghent University, Ottergemsesteenweg 460, Ghent 9000, Belgium; Faculty of Medicine and Health Sciences, Department of Internal Medicine and Pediatrics, Ghent University Hospital, Corneel Heymanslaan 10, Ghent 9000, Belgium; Faculty of Medicine and Health Sciences, Department of Medical Oncology, Ghent University Hospital, Corneel Heymanslaan 10, Ghent 9000, Belgium; Faculty of Pharmaceutical Sciences, Department of Bio-analysis, Pharmaceutical Care Unit, Ghent University, Ottergemsesteenweg 460, Ghent 9000, Belgium

**Keywords:** Bleeding, Tumoural lesion, Cancer, Early detection, Oral anticoagulant, Atrial fibrillation

## Abstract

**Aims:**

Bleeding events are a well-known complication of oral anticoagulant (OAC) use in patients with atrial fibrillation (AF). While these are undesirable, bleedings could have a warning potential for underlying tumoural lesions. Therefore, we aimed to investigate the association between anticoagulant-related bleeding and newly diagnosed tumoural lesions in a nationwide cohort study.

**Methods and results:**

Using Belgian nationwide data, AF patients without any tumoural lesions were included when initiating OACs between 2013 and 2019. The absolute and relative risks of newly diagnosed tumoural lesions were investigated in OAC users with vs. without an OAC-related bleeding event. Analyses were additionally stratified by tumoural lesion, location-specific bleeding, and OAC type. A total of 230 386 OAC users were included, among whom 35 192 persons were diagnosed with a tumoural lesion during follow-up. Persons with a clinically relevant bleeding during OAC use had a tumoural lesion incidence of 15.33 per 100 person-years compared to an incidence of 5.22 per 100 person-years in persons without bleeding. Site-specific gastrointestinal, urogenital, respiratory, and intracranial bleeding events were respectively associated with a significantly increased risk of incident gastrointestinal [adjusted hazard ratio (aHR) 8.13 (95% confidence interval (CI): 7.08–9.34)], urological [aHR 12.73 (95% CI: 10.56–15.35)], respiratory [aHR 4.91 (95% CI: 3.24–7.44)], and intracranial tumoural lesions [aHR 27.89 (95% CI: 16.53–47.04)].

**Conclusion:**

Bleeding events in AF patients initiated on OAC were associated with an increased risk of tumoural lesions. Therefore, OAC-related bleeding events could unmask an underlying tumoural lesion.

## Graphical abstract

**Graphical abstract:**
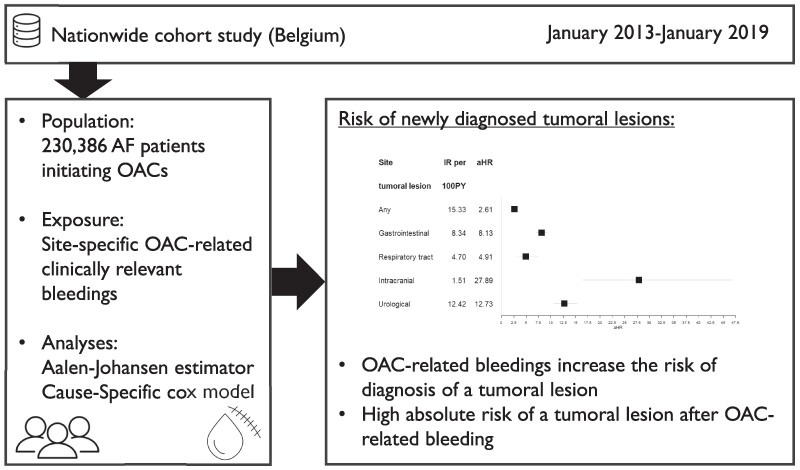

## Introduction

The global oral anticoagulant (OAC) use almost doubled between the periods of 2010 and 2018.^1^ Oral anticoagulants have become a pillar in the primary therapeutic strategy for stroke prevention in patients with atrial fibrillation (AF).^2,3^ Currently, two classes of OACs are used, namely vitamin K antagonists (VKAs) and non-vitamin K antagonist oral anticoagulants (NOACs). However, OACs are associated with a considerably increased risk of bleeding, especially intracranial bleeding with VKAs and gastrointestinal bleeding with NOACs.^4–11^

While bleeding events are an undesirable side effect of anticoagulants in AF patients, these could have a warning potential. In the general population, major bleeding episodes are indeed well-established markers for underlying tumoural lesions.^12–14^ Likewise, gastrointestinal bleeding has been associated with newly diagnosed tumoural lesions among OAC users, albeit studies were small or only based on case reports.^15–18^

Therefore, we aimed to investigate the association between anticoagulant-related bleeding and newly diagnosed tumoural lesions in AF patients in a nationwide cohort study. Absolute rates and relative risks of newly diagnosed tumoural lesions were investigated in OAC users with vs. without a precipitating OAC-related bleeding event.

## Methods

### Data source

Details on the study methodology have been published before.^19^ In brief, two nationwide databases were used, namely the InterMutualistic Agency (IMA) database and Minimal Hospital Dataset (MHD). The IMA centralizes all claims data from Belgian health insurance funds on reimbursed ambulatory and hospital care, including demographic characteristics, medical procedures, and drug prescription claims, and represents all legal residents in Belgium.^20^ The MHD aggregates all hospital discharge diagnoses (hospitalizations, day-care stays, and emergency room contacts), coded in International Classification of Diseases (ICD) codes (ICD-9 up to 2014, ICD-10 from 2015 onwards).^21^ All single cases of the study population were included in both databases and could be identified. This study was approved by the Belgian Commission for the Protection of Privacy (approval code IVC/KSZG/20/344).^22^ The Strengthening the Reporting of Observational Studies in Epidemiology (STROBE) reporting guideline was followed (see Supplementary material online, *Table S1*).^23^

### Study population

Subjects ≥ 45 years old with ≥1 year coverage by Belgian health insurance funds were included on the first date of filling an OAC prescription (=index date) from 1 January 2013 to 1 January 2019. Vitamin K antagonists (warfarin, acenocoumarol, or phenprocoumon) and NOAC users (dabigatran, rivaroxaban, apixaban, or edoxaban) were included. Only OAC-naïve subjects eligible for NOAC and VKA were considered. Patients with a contraindication for NOAC or VKA as described in previous studies, and people with a diagnosed tumoural lesion ≤ 1 year before the index date, were excluded (see Supplementary material online, *Table S2*, Supplementary material online, *Figure S1*).^19,24^

### Covariables

Baseline characteristics were defined based on medical procedure codes, ICD-coded diagnoses, and/or medication prescription claims within one year before the index date. Medication history was identified with medication prescription claims, considering recent use ≤6 months before the index date (see Supplementary material online, *Figure S1*, Supplementary material online, *Table S2*). The covariables at baseline were selected based on risk factors for bleeding events or cancer in previous research (see Supplementary material online, *Table S2*).^25–31^

The CHA_2_DS_2_-VASc score, modified HAS-BLED score (excluding the ‘labile INR’ criterion), the Johns Hopkins Claims-based Frailty Indicator (CFI), frailty score (based on the frailty the Claims-based Frailty Indicator), and age-specific Charlson comorbidity index were calculated.^32–35^

### Outcomes and exposure

The primary outcome was defined as any incident diagnosis of a tumoural lesion after OAC initiation. As a secondary outcome, site-specific tumoural lesions were investigated separately, namely in the gastrointestinal, intracranial, respiratory tract, and urinary tract (including kidney, ureter, bladder, prostate, and urethra tumoural lesions) region. Outcomes were identified using ICD-coded hospital discharge diagnoses (e.g. ICD-10 code C34.9 for a tumoural lesion in the respiratory tract) and medical procedure codes (see Supplementary material online, *Table S3*). Additionally, a supplementary analysis was performed focusing on haematological cancers.

The exposure of interest was (1) a major or clinically relevant non-major bleeding (MB/CRNMB) event occurring after OAC initiation but preceding any diagnosis of a tumoural lesion, and (2) the type of OAC related to the major or clinically relevant non-major bleeding (MB/CRNMB) event. Major bleeding was defined as a hospitalized bleeding event in a critical area or organ (e.g. intracranial), fatal bleeding or bleeding event with a medical procedure code for blood transfusion ≤ 10 days after admission, which is adapted from the International Society on Thrombosis and Haemostasis definition due to a lack of data on haemoglobin levels or number of blood transfusion units.^36,37^ Clinically relevant non-major bleeding was defined as a bleeding event requiring hospitalization that did not classify for major bleeding. Bleeding events were identified using ICD-coded hospital discharge diagnoses and specific medical procedure codes, and were additionally classified by site-specific bleeding (e.g. intracranial bleeding). Patients with major bleedings or CRNMB could have multiple bleedings at different locations. The type of OAC use was identified with drug prescription claims and divided into two categories: VKAs (warfarin, phenprocoumon, and acenocoumarol) and NOACs (dabigatran, rivaroxaban, apixaban, and edoxaban).

### Follow-up

Follow-up started at the onset of OAC initiation until one of the following events occurred: outcome of interest, death, emigration, or end of study period (1 January 2019), whichever came first.

### Statistical analysis

Descriptive statistics were used to describe the overall study population. Continuous variables were described by median and interquartile range (IQR). Categorical variables were shown as counts (*n*) with percentages (%).

The absolute risk of a newly diagnosed tumoural lesion in patients with vs. without a preceding OAC-related bleeding event was described by a cumulative incidence function, derived by an Aalen–Johansen estimator accounting for the competing risk of death.^38^ The relative risk of newly diagnosed tumoural lesions was estimated with a cause-specific Cox model, incorporating the occurrence of a bleeding event as a time-dependent covariate.^39^ Adjusted hazard ratios (aHRs) with 95% confidence intervals (95% CIs) were calculated. The model was multivariable adjusted for cancer- and bleeding-related covariables described in *Table 1* (e.g. age and sex). An interaction term between the type of OAC and bleeding was taken into account to check for heterogeneity between different OAC-related bleeding events. The proportional hazards assumption was checked based on plots of the scaled Schoenfeld residuals over time.^40^ A two-sided *P*-value of <0.05 was considered statistically significant. All analyses were performed in R software (R^®^; version 4.2.3; Vienna, Austria).

**Table 1:** Baseline characteristics of the study population

	Variable	*n* = 230 386
Demographics	Age (year), mean (SD)	74.38 (10.96)
	Female sex, *n* (%)	110 729 (48.06)
OAC	VKA, *n* (%)	55 805 (24.22)
	NOAC, *n* (%)	174 581 (75.78)
Tumoural lesion	Any tumoural lesion, *n* (%)	35 192 (15.28)
	Gastrointestinal, *n* (%)	6900 (2.99)
	Respiratory tract, *n* (%)	2217 (0.96)
	Intracranial, *n* (%)	469 (0.20)
	Urologic, *n* (%)	4383 (1.9)
Bleedings	Major bleed or CRNMB, *n* (%)	26 920 (11.68)
	Major bleed, *n* (%)	19 493 (8.46)
	CRNMB, *n* (%)	9901 (4.30)
	Gastrointestinal, *n* (%)	11 477 (4.98)
	Respiratory tract, *n* (%)	1875 (0.81)
	Intracranial, *n* (%)	3250 (1.41)
	Urogenital, *n* (%)	5253 (2.28)
Covariates	Hypertension, *n* (%)	146 939 (63.78)
	Coronary artery disease, *n* (%)	40 255 (17.47)
	Peripheral artery disease, *n* (%)	16 092 (6.98)
	Dyslipidaemia, *n* (%)	130 482 (56.64)
	Chronic kidney disease, *n* (%)	23 011 (9.99)
	Chronic liver disease, *n* (%)	5231 (2.27)
	Chronic lung disease, *n* (%)	37 144 (16.12)
	Pneumonia, *n* (%)	10 946 (4.75)
	Upper GI tract disorder^a^, *n* (%)	14 222 (6.17)
	Lower GI tract disorder^b^, *n* (%)	12 246 (5.32)
	Inflammatory bowel disease, *n* (%)	787 (0.34)
	Diabetes mellitus, *n* (%)	51 830 (22.5)
	Anaemia, *n* (%)	14 092 (6.12)
Medication	Drugs at baseline, mean (SD)	6.45 (4.07)
	NSAID, *n* (%)	55 382 (24.04)
Risk scores	CHA_2_DS_2_-VASc score, median (Q1–Q3)	3.00 (2.00–4.00)
	HAS-BLED score, median (Q1–Q3)	2.00 (2.00–3.00)
	Charlson comorbidity index, median (Q1–Q3)	4.00 (3.00–5.00)
	Frailty score, median (Q1–Q3)	0.16 (0.05–0.21)

CRNMB, clinically relevant non-major bleeding; GI, gastrointestinal; NOAC, non-vitamin K antagonist oral anticoagulant; NSAID, non-steroidal anti-inflammatory drug; OAC, oral anticoagulant; SE, systemic embolism; SD, standard deviation; VKA, vitamin K antagonist. ^a^Upper gastrointestinal tract disorders were defined as gastroesophageal reflux diseases or peptic ulcer disease.

^b^Lower gastrointestinal tract disorder was defined as diverticulosis, angiodysplasia, colorectal polyposis, or haemorrhoids. Since patients could have multiple tumoural lesions or multiple bleedings at different locations, numbers of region-specific lesions and bleedings do not sum up to the total number of patients with any tumoural lesion or major bleed or CRNMB, respectively.

Analyses were repeated for a site-specific subgroup analysis, investigating the site-specific risk of a tumoural lesion for a local bleeding (e.g. tumoural lesion in gastrointestinal region after a gastrointestinal bleeding). Newly diagnosed lesions on a different site were considered a competing risk. In case of multiple diagnoses of tumoural lesions on the same day, the lesion at the site of interest was considered the outcome. Furthermore, a distinction was made between an initial bleeding occurring within 6 months after OAC initiation and a bleeding after 6 months.

### Sensitivity analysis

As a sensitivity analysis, a nested case–control study was performed using multivariable conditional logistic regression models, including the same covariables as the cause-specific Cox model. A person with a tumoural lesion diagnosis within the period of interest was considered a ‘case’. This case was matched by risk set sampling, with a matching ratio of 1:4. The matching factors were age (±1year), sex, and follow-up time.

## Results

### Study population

The study included 230 386 OAC-treated AF patients during a median follow-up of 3.28 years (IQR: 1.86–4.94 years). There were slightly more men (51.94%), and the mean age was 74.38 years (IQR: 67.00–83.00 years) (*Table 1*). During the follow-up period, 35 192 subjects (15.28%) were newly diagnosed with a tumoural lesion, among whom 15.40% were diagnosed after an OAC-related bleeding.

### Risk of a tumoural lesion after bleeding

**Figure 1:**
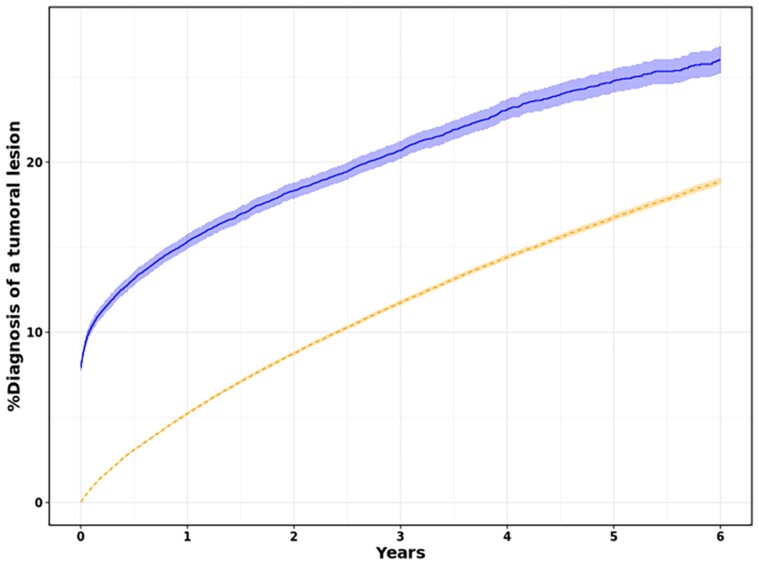
Cumulative incidence function for a diagnosis of a tumoural lesion following oral anticoagulant (OAC) initiation in atrial fibrillation patients, stratified according to the experience of a bleeding event. The follow-up started at the onset of an OAC-related bleeding (solid line), whereas at the time of OAC initiation in case no bleeding occurred (dashed line).

The absolute risk of newly diagnosed tumoural lesions among OAC users, stratified by whether or not a major bleeding or a CRNMB occurred, is shown in *Figure 1*. For OAC users who did not experience such a bleeding, the estimated 100 person-years incidence was 5.22 (95% CI: 5.12–5.32). In contrast, after a bleeding event, the 100 person-years incidence was 15.33 (95% CI: 14.90–15.77).

The estimated relative risk for a subsequent tumoural lesion diagnosis after a major bleeding or a CRNMB is shown in *Table 2*. Oral anticoagulant users who experienced an MB/CRNMB had a 2.61-fold higher risk for an incident diagnosis of a tumoural lesion following an OAC-related bleeding event [aHR 2.61 (95% CI: 2.46–2.77)]. Results were consistent when it was stratified between the first 6 months after the start of OAC initiation [aHR 2.25 (95% CI: 2.10–2.42)] and bleedings events occurring later after the OAC initiation [aHR 2.88 (95% CI: 2.69–3.07)]. Compared to VKA-related bleedings, NOAC-related bleedings had an additionally increased risk of 1.13 (95% CI: 1.06–1.20).

**Table 2:** A multivariate cause-specific Cox regression on the risk of an incident tumoural lesion, among OAC users with vs. without a bleeding event

Variables	Tumoural lesion aHR (95% CI)
Bleeding effect (MB/CRNMB)	
Bleeding after OAC initiation	2.61 (2.46–2.77)
Type OAC related to bleeding (ref = VKA)	
NOAC-related bleeding	1.13 (1.06–1.21)
Demographics	
Age	1.03 (1.03–1.03)
Sex	0.70 (0.68–0.72)
Comorbidities	
Hypertension	0.96 (0.93–0.99)
CAD	0.94 (0.91–0.98)
Peripheral artery disease	1.13 (1.08–1.19)
Dyslipidaemia	0.95 (0.92–0.97)
Chronic kidney disease	1.03 (0.99–1.08)
Chronic liver disease	1.16 (1.08–1.25)
Chronic lung disease	1.18 (1.14–1.22)
Pneumonia	1.06 (1.01–1.12)
Upper GI tract disorder^a^	1.01 (0.97–1.06)
Lower GI tract disorder^b^	1.14 (1.09–1.20)
Inflammatory bowel disease	1.21 (1.01–1.45)
Diabetes mellitus	1.00 (0.97–1.03)
Anaemia	1.09 (1.04–1.15)
Comorbidity scores	
CCI	0.99 (0.97–1.00)
CHA_2_DS_2_-VASc score	0.99 (0.98–1.01)
Frailty score	0.34 (0.30–0.39)
HAS-BLED score	1.03 (1.01–1.05)
Medication usage	
Drug number at baseline	1.02 (1.02–1.03)
NSAID	0.99 (0.96–1.01)

aHR, adjusted hazard ratio; CCI, Charlson comorbidity index; CI, confidence interval; CRNMB, clinically relevant non-major bleeding; GI, gastrointestinal; MB, major bleeding; NOAC, non-vitamin K antagonist oral anticoagulant; NSAID, non-steroidal anti-inflammatory drug; OAC, oral anticoagulant; SE, systemic embolism; SD, standard deviation; VKA, vitamin K antagonist.

^a^Upper gastrointestinal tract disorders were defined as gastroesophageal reflux diseases or peptic ulcer disease.

^b^Lower gastrointestinal tract disorder was defined as diverticulosis, angiodysplasia, colorectal polyposis, or haemorrhoids.

### Site-specific risk of a tumoural lesion

**Figure 2:**
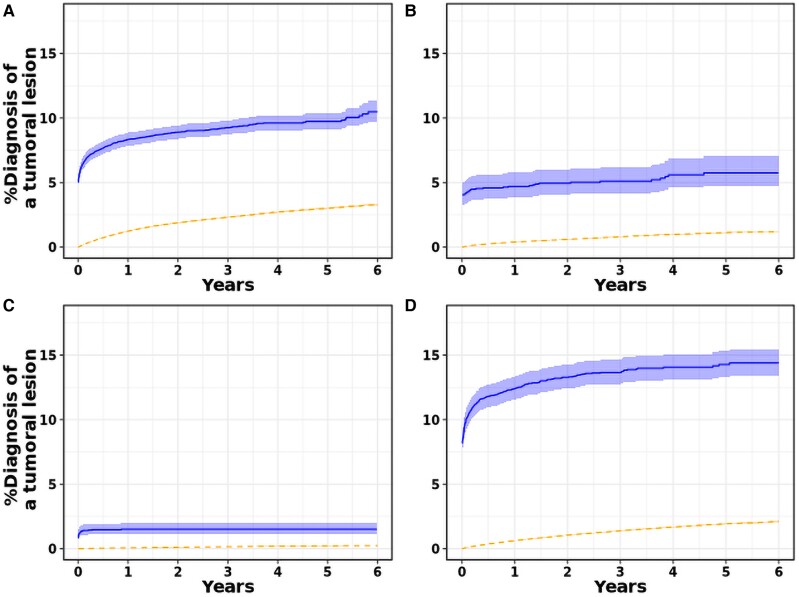
The absolute risk of a tumoural lesion diagnosis in the (*A*) gastrointestinal, (*B*) respiratory tract, (*C*) intracranial, (*D*) urological region following oral anticoagulant (OAC) initiation in atrial fibrillation patients, stratified according to the experience of a site-specific bleeding event. The follow-up started at the onset of an OAC-related bleeding (solid line), whereas at the time of OAC initiation in case no bleeding occurred (dashed line).

A subgroup analysis of the absolute risk of a tumoural lesion stratified by site is shown in *Figure 2*; the absolute risk of a haematological malignancy is shown in Supplementary material online, *Figure S2*. A local bleeding increased the risk of a site-specific tumoural lesion diagnosis significantly, especially the risk of gastrointestinal and urological lesions, with the diagnosis mainly being established shortly after a bleeding event.

The relative risk of the incident diagnosis of a region-specific tumoural lesion is shown in *Table 3*. The relative risk for haematological cancers is shown in Supplementary material online, *Table S4*. All regions showed an increased risk after corresponding location-specific bleeding events. The highest increase was found in the intracranial region [aHR 27.89 (95% CI: 16.53–47.04)]. In the case of gastrointestinal, respiratory tract, and urological tract bleeding events, an 8.13−, 4.91−, and 12.73-fold higher risk was observed, respectively. Remarkably, there was also an increased risk of a tumoural lesion diagnosis after the occurrence of a non-local bleeding.

**Table 3:** A multivariable cause-specific Cox regression on the risk of an incident tumoural lesion diagnosis, among OAC users, stratified by site-specific bleeding and tumoural lesion

Hazard ratio	Gastrointestinal tumoural lesion	Tumoural lesion In respiratory tract	Intracranial tumoural lesion	Tumoural lesion in urological tract
	aHR (95% CI)	aHR (95% CI)	aHR (95% CI)	aHR (95% CI)
Local bleeding event				
Bleeding after OAC initiation	8.13 (7.08–9.34)	4.91 (3.24–7.44)	27.89 (16.53–47.04)	12.73 (10.56–15.35)
Type of OAC related to bleeding (ref = VKA)				
NOAC-related bleeding	1.04 (0.89–1.21)	2.11 (1.31–3.40)	0.84 (0.45–1.55)	0.93 (0.76–1.14)
Demographics				
Age	1.02 (1.02–1.03)	1.02 (1.02–1.03)	1.01 (0.99–1.02)	1.06 (1.05–1.06)
Sex	0.78 (0.73–0.83)	0.46 (0.40–0.52)	0.78 (0.6–1.02)	0.20 (0.18–0.22)
Comorbidities				
Hypertension	0.95 (0.88–1.02)	0.84 (0.74–0.96)	1.04 (0.79–1.36)	0.98 (0.9–1.07)
CAD	0.95 (0.88–1.03)	0.88 (0.77–1.00)	0.80 (0.58–1.10)	1.01 (0.92–1.11)
Peripheral artery disease (smoking)	1.11 (1–1.23)	1.68 (1.44–1.96)	1.40 (0.98–2.01)	1.15 (1.01–1.31)
Dyslipidaemia	1.00 (0.94–1.05)	0.90 (0.82–0.99)	1.12 (0.91–1.37)	1.00 (0.94–1.07)
CKD	0.90 (0.82–1)	0.82 (0.68–0.99)	0.68 (0.43–1.07)	1.08 (0.96–1.21)
Chronic liver disease	1.14 (0.97–1.35)	0.82 (0.55–1.22)	0.78 (0.35–1.74)	0.87 (0.68–1.11)
Chronic lung disease	1.25 (1.16–1.34)	1.87 (1.66–2.09)	1.55 (1.17–2.06)	1.07 (0.98–1.18)
Pneumonia	1.04 (0.92–1.17)	1.48 (1.24–1.77)	1.12 (0.66–1.89)	0.93 (0.78–1.11)
Upper GI tract disorder^a^	1.10 (1.00–1.22)	0.86 (0.72–1.03)	0.77 (0.48–1.23)	0.9 (0.78–1.03)
Lower GI tract disorder^b^	1.35 (1.23–1.49)	0.92 (0.76–1.11)	1.00 (0.65–1.54)	1.05 (0.92–1.2)
Inflammatory bowel disease	1.07 (0.72–1.60)	0.73 (0.37–1.45)	1.22 (0.29–5.11)	1.40 (0.76–2.58)
Diabetes mellitus	1.10 (1.03–1.18)	0.85 (0.75–0.96)	0.83 (0.63–1.09)	1.00 (0.91–1.09)
Anaemia	1.07 (0.96–1.20)	0.90 (0.75–1.09)	1.39 (0.90–2.13)	1.02 (0.88–1.17)
Risk scores				
CCI	0.98 (0.95–1.02)	1.05 (0.99–1.11)	1.03 (0.90–1.17)	0.99 (0.95–1.03)
CHA_2_DS_2_-VASc score	1.00 (0.96–1.03)	0.95 (0.89–1.02)	0.97 (0.82–1.15)	0.97 (0.92–1.02)
Frailty score	0.24 (0.18–0.33)	0.29 (0.17–0.50)	0.18 (0.05–0.63)	0.40 (0.28–0.58)
HAS-BLED score	1.04 (1.00–1.09)	1.13 (1.05–1.21)	1.03 (0.88–1.22)	1.03 (0.98–1.09)
Medication usage				
Drug number at baseline	1.02 (1.01–1.02)	1.03 (1.02–1.05)	1.00 (0.97–1.03)	1.03 (1.01–1.04)
NSAID	0.98 (0.92–1.04)	0.88 (0.79–0.97)	0.95 (0.75–1.20)	0.97 (0.90–1.05)

CCI, Charlson comorbidity index; CRNMB, clinically relevant non-major bleeding; MB, major bleeding; NOAC, non-vitamin K antagonist oral anticoagulant; NSAID, non-steroidal anti-inflammatory drug; OAC, oral anticoagulant; SE, systemic embolism; VKA, vitamin K antagonist.

^a^Upper gastrointestinal tract disorders were defined as gastroesophageal reflux diseases or peptic ulcer disease.

^b^Lower gastrointestinal tract disorder was defined as diverticulosis, angiodysplasia, colorectal polyposis, or haemorrhoids.

Only in the case of respiratory tract tumoural lesions, a significantly higher risk after respiratory tract bleeding was observed in NOAC- as compared to VKA-related bleeding events [aHR_interaction_: 2.11 (95% CI: 1.31–3.40)].

### Sensitivity analyses

The nested case–control study confirmed the significant increase in risk for a subsequent diagnosis of a tumoural lesion among people who experienced a bleeding [aHR 2.61 (95% CI: 2.43–2.79)]. Furthermore, a significant interaction between the type of OAC and a clinical bleeding on the risk of a tumoural lesion was observed [aHR_interaction_: 1.10 (95% CI: 1.02–1.19)] (*Table 4*).

**Table 4:** A multivariate logistic regression on the risk of a tumoural lesion among OAC users

Odds ratio	Any tumoural lesion	Gastrointestinal tumoural lesion	Tumoural lesion in respiratory tract	Intracranial tumoural lesion	Tumoural lesion in urological tract
	OR (95% CI)	OR (95% CI)	OR (95% CI)	OR (95% CI)	OR (95% CI)
Local bleeding event					
MB/CRNMB	2.61 (2.43–2.79)	/	/	/	/
Gastrointestinal bleeding	/	7.16 (5.46–9.40)	2.54 (1.41–4.56)	0.92 (0.18–4.84)	1.78 (1.11–2.85)
Respiratory tract bleeding	/	1.16 (0.61–2.22)	5.11 (2.34–11.17)	2.29 (0.11–46.64)	1.68 (0.7–3.99)
Intracranial bleeding	/	1.57 (0.71–3.48)	4.32 (1.41–13.27)	31.82 (5.92–171.15)	0.80 (0.29–2.22)
Urological bleeding	/	1.48 (0.86–2.55)	1.96 (0.75–5.14)	2.75 (0.37–20.62)	12.29 (8.17–18.51)
Type OAC related to bleeding (ref = VKA)					
MB/CRNMB	1.10 (1.02–1.19)	/	/	/	/
Gastrointestinal bleeding	/	1.21 (0.89–1.66)	1.32 (0.67–2.59)	1.48 (0.24–9.18)	1.26 (0.74–2.13)
Respiratory tract bleeding	/	0.99 (0.43–2.26)	2.09 (0.77–5.65)	1.67 (0.06–44.32)	0.67 (0.23–1.89)
Intracranial bleeding	/	0.92 (0.34–2.43)	0.56 (0.15–2.06)	1.11 (0.14–8.52)	2.44 (0.77–7.68)
Urological bleeding	/	0.93 (0.50–1.73)	1.22 (0.43–3.48)	2.44 (0.24–25.29)	0.89 (0.57–1.39)
Comorbidities					
Hypertension	1.00 (0.96–1.04)	1.01 (0.91–1.12)	0.89 (0.74–1.07)	1.15 (0.78–1.70)	0.95 (0.83–1.09)
CAD	0.97 (0.93–1.01)	0.97 (0.87–1.08)	0.80 (0.67–0.97)	0.80 (0.51–1.26)	0.95 (0.83–1.09)
Peripheral artery disease	1.24 (1.18–1.31)	1.17 (1.00–1.36)	1.63 (1.3–2.03)	1.55 (0.89–2.70)	1.21 (0.99–1.48)
Dyslipidaemia	0.94 (0.91–0.96)	1.00 (0.93–1.07)	0.93 (0.82–1.05)	1.15 (0.87–1.52)	0.98 (0.89–1.07)
Chronic kidney disease	1.09 (1.04–1.15)	1.01 (0.88–1.15)	0.92 (0.71–1.19)	0.77 (0.39–1.52)	1.13 (0.96–1.33)
Chronic liver disease	1.43 (1.31–1.56)	1.32 (1.03–1.68)	0.91 (0.51–1.64)	1.25 (0.43–3.62)	0.80 (0.54–1.19)
Chronic lung disease	1.23 (1.18–1.27)	1.22 (1.11–1.35)	1.84 (1.56–2.16)	1.17 (0.80–1.70)	1.20 (1.05–1.37)
Pneumonia	1.12 (1.05–1.19)	1.03 (0.87–1.22)	1.47 (1.13–1.91)	1.59 (0.81–3.13)	0.84 (0.67–1.05)
Upper GI tract disorder^a^	1.09 (1.03–1.14)	1.23 (1.08–1.41)	0.91 (0.71–1.18)	0.72 (0.37–1.39)	0.81 (0.67–0.98)
Lower GI tract disorder^b^	1.15 (1.09–1.21)	1.54 (1.35–1.76)	0.93 (0.73–1.20)	0.67 (0.38–1.19)	0.98 (0.82–1.17)
Inflammatory bowel disease	1.24 (1.02–1.52)	0.91 (0.51–1.64)	0.76 (0.30–1.92)	3.15 (0.29–34.07)	1.28 (0.59–2.80)
Diabetes mellitus	1.04 (1.00–1.07)	1.08 (0.98–1.18)	0.84 (0.71–0.99)	0.79 (0.53–1.19)	1.00 (0.88–1.15)
Anaemia	1.16 (1.10–1.23)	1.18 (1.01–1.38)	1.10 (0.83–1.45)	1.44 (0.75–2.78)	1.03 (0.83–1.27)
Risk score					
CCI	0.94 (0.92–0.96)	0.96 (0.91–1.01)	1.04 (0.93–1.15)	0.98 (0.80–1.21)	0.96 (0.90–1.03)
CHA_2_DS_2_-VASc score	1.00 (0.97–1.02)	1.02 (0.96–1.09)	1.00 (0.89–1.12)	0.95 (0.75–1.20)	0.97 (0.89–1.06)
Frailty score	0.30 (0.25–0.35)	0.49 (0.32–0.75)	0.49 (0.22–1.09)	0.81 (0.11–6.10)	0.74 (0.42–1.30)
HAS-BLED score	0.98 (0.96–1.01)	0.91 (0.85–0.96)	1.04 (0.93–1.16)	0.95 (0.73–1.25)	1.03 (0.95–1.11)
Medication usage					
Drug number at baseline	1.10 (1.02–1.19)	1.02 (1.01–1.03)	1.03 (1.01–1.05)	1.00 (0.96–1.05)	1.03 (1.01–1.04)
NSAID	1.01 (0.98–1.04)	1.04 (0.96–1.13)	0.94 (0.82–1.09)	0.92 (0.66–1.28)	1.00 (0.90–1.11)

CCI, Charlson comorbidity index; CI, confidence interval; CRNMB, clinically relevant non-major bleeding; GI, gastrointestinal; MB, major bleeding; NOAC, non-vitamin K antagonist oral anticoagulant; NSAID, non-steroidal anti-inflammatory drug; OAC, oral anticoagulant; OR, odds ratio; SE, systemic embolism; VKA, vitamin K antagonist.

^a^Upper gastrointestinal tract disorders were defined as gastroesophageal reflux diseases or peptic ulcer disease.

^b^Lower gastrointestinal tract disorder was defined as diverticulosis, angiodysplasia, colorectal polyposis, or haemorrhoids.

## Discussion

Oral anticoagulant therapy interferes with common haemostatic processes, leading to adverse bleeding events, especially in case of underlying pre-existing lesions (e.g. colorectal polyposis).^41–44^ While bleeding events are an undesired side effect of OACs, they could have the potential to unveil underlying malignancies, enabling an earlier diagnosis and subsequent treatment.^15,45,46^ Indeed, in this study, we have demonstrated that AF patients who suffered a clinical relevant bleeding after OAC initiation, had a one-year risk of a newly diagnosed tumoural lesion of 15%, as compared to 5% among OAC users without any bleeding event. Therefore, subjects with a bleeding event while being treated with OACs may benefit from an intensive diagnostic work-up to unveil underlying tumoural lesions.

Our results are in line with previous research that also demonstrated increased risks of tumoural lesions following OAC-related bleeding events.^13,16,47^ Exemplary, in a recent meta-analysis, anticoagulant-related bleeding events were associated with a 6.1- and 15.2-fold increased odds of tumour detection in AF patients treated with NOACs and VKAs, respectively.^16^ The impact of the type of OAC related to the bleeding on tumoural lesions is still inconclusive.^16,48^ Our results did show a higher risk of an incident tumoural lesion diagnosis after a NOAC-related bleeding compared to VKA-related bleedings. A possible explanation may be differences in underlying baseline characteristics between NOAC users and VKA users.^9–11^ In Belgium, NOACs are more commonly initiated in AF patients than VKAs, and tend to be more prescribed in older geriatric AF patients with age-associated traits (e.g. frailty), whereas VKAs were more frequently initiated in patients with cardiovascular, renal, and hepatic comorbidities.^1,49^ Hence, the a prior probability of cancer in NOAC users may be higher due to unmeasured confounding in older geriatric AF patients.^9^ Furthermore, the risk of a non-cancerous bleeding aetiology may be higher in VKA users, due to the higher prevalence of cardiovascular, renal, and hepatic comorbidities. While higher risks for gastrointestinal bleeding have been reported in specific types of NOACs compared to VKA, the general risk of clinically relevant bleedings remains lower, mainly driven by a lower risk of intracranial bleeding.^36^

In the present study, the risk of diagnosing underlying tumoural lesions was especially increased in case of a preceding bleeding event in the gastrointestinal or urological tract. In the general population, gastrointestinal bleeding is a well-known symptom of underlying tumoural lesions.^50–52^ As OAC users are at an increased risk of gastrointestinal bleeding, especially NOACs users, tumoural lesions in the GI tract, mostly related to primary tumours in the GI tract, may be detected earlier.^36^ Exemplary, a recent study showed that lower gastrointestinal bleeding events have four times higher odds of being provoked by colorectal cancers in OAC users compared to non-users.^41^ Likewise, 3.7–8.1% (depending on age) and 8.06% of gastrointestinal bleeding events were associated with malignancy in a recent nationwide cohort study and *post hoc* analysis of the RE-LY trial, respectively.^17,53^ In our study, AF patients with gastrointestinal bleeding after OAC initiation had an 8.13-fold higher risk of gastrointestinal tumoural lesions compared to OAC-treated AF patients without a gastrointestinal bleeding event. Therefore gastrointestinal bleedings should be taken seriously as a potential sign of a tumoural lesion.

As urogenital bleeding (haematuria) is also associated with urological tumours,^54–56^ several guidelines have argued in favour of screening after haematuria.^57,58^ As the risk of haematuria is increased among OAC users,^59^ our study together with a Danish nationwide study demonstrated a strong association between urogenital bleeding events and an increased risk of tumoural lesions in the urinary tract among OAC users.^60^

In our nested case–control study, we also observed an association between site-specific tumoural lesions and non-local bleedings. This could reflect metastasis in other regions, invasive growth, or cancer-related interference in haemostasis or intestinal mucosa.^61^

Surprisingly, a higher frailty score was related to a lower risk of an incident tumoural lesion. This is mainly driven by the competing risk of mortality. During follow-up, 37.91% of the frail people (frailty score > 0.2) died. In contrast, 8.83% of the non-frail people died.

Previous research indicated delays in tumoural diagnoses among people who suffer from a bleeding episode.^62^ Unfortunately, data describing the delay between the first occurrence of symptoms to medical help-seeking and the final cancer diagnosis have not been recorded for this study. We strongly recommend future research, investigating intervals from the first indication to help-seeking and the cancer diagnosis among people experiencing a bleeding and people who have experienced other indications.

Our results support the notion that bleedings within the anticoagulated population are a strong marker of malignancies, rather than solely a consequence of OAC treatment.^15,18,60,63^ It is plausible that OAC usage could promote bleedings to an extent that it becomes clinically visible, which could facilitate an early tumour diagnosis. However, future research on the aetiology of OAC-related bleedings will be essential to confirm this hypothesis. Based on our results and the existing studies on OAC-related bleedings, a careful evaluation should be made on the benefits of extensive work-up for an eventual cancer diagnosis after a bleeding event compared to the associated risks and costs of these procedures.^64^ A follow-up study on the stage of the detected tumoural lesion shortly after a bleeding event and potential savings on the outcome of these early uncovered tumoural lesions could give a better understanding of the benefits of possible screening interventions.

## Strengths and limitations

We contributed to an area of research that is limited to a small number of studies. A major strength is the large cohort of unselected OAC users. Furthermore, we incorporated a broad range of comorbidities and accounted for the competing risk of death. Additionally, our cohort contains mainly patients on NOAC, which is becoming the standard of care in AF patients over the last years.^1,65^

However, our study has several limitations. First, in case of a hospitalization, the diagnosis will be reported at the time of discharge. In case of a bleeding and a diagnosis of a tumoural lesion during the same hospitalization, it was impossible to retrace whether the bleeding occurred before the diagnosis of the tumoural lesion, or if it was a post-interventional bleeding. Therefore the initial risk of a tumoural lesion diagnosis might be elevated due to bleeding occurring during the diagnostic work-up for cancer.

Second, due to the observational design, we cannot infer causation. However, there are possible mechanisms explaining why these bleeding events may be informative for tumoural lesions.

Third, there could be an underestimation of underlying incidence of tumoural lesions among non-bleeders, as bleeding events could have prompted a hospitalisation and subsequent diagnostic work-up to discover underlying lesions. This may not have been the case in asymptomatic or mildly symptomatic cancer patients without bleeding symptoms (e.g. only minor weight loss or constipation not leading to a healthcare contact).

Fourth, certain lifestyle characteristics were missing like weight, socioeconomic status, and smoking. These variables could have important confounding effects as BMI and smoking both influence the bleeding risk and cancer risk. Socioeconomic status could have a mediation effect as there could be differences in the medical consultation and therapy adherence to OACs.

Fifth, our study investigated the risk of incident tumoural lesions. However, our data lack the definitive cancer diagnosis. To get the definitive cancer diagnosis, pathology reports are required. These definitive cancer diagnoses could give insight into whether these bleedings are mainly associated with the primary tumour. Moreover, further insight could be provided into the aetiology of these bleedings. Furthermore, several persons’ initial codes were not specific enough to be classified or excluded as one of the categories of interest (*n* = 9917; 28%). Therefore, the absolute risk of the tumoural lesions in the subgroup might be underestimated.

Lastly, we only had data on bleeding events that resulted in a hospitalisation. Minor bleedings that did not require a hospitalization could also be relevant markers for a possible tumour.

## Conclusion

This nationwide cohort showed that local bleeding events among OAC users are associated with a high risk of newly diagnosed tumoural lesions, especially tumoural lesions in the gastrointestinal and urogenital region. Our results support the notion that bleedings within the anticoagulated population are a strong marker of malignancies. An evaluation of clinical guidelines for bleeding among OAC users could benefit early detection of tumoural lesions.

## Lead author biography


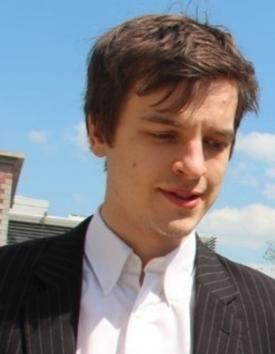


Kristiaan Proesmans is a public health researcher. He received a degree in biomedical engineering and statistical data analyses in Belgium (University of Ghent). Following, he spent three years at Sciensano, the Belgium federal institute for public health, focusing on the epidemiology of infectious diseases. In 2023, Kristiaan advanced his academic career by joining the Faculty of Pharmaceutical Sciences at the University of Ghent, where he is currently pursuing a Ph.D. in the Department of Bio-Analyses. Throughout his career, he has contributed to several projects and scientific publications focused on public health and epidemiology.

## Supplementary Material

oeae081_Supplementary_Data

## Data Availability

Requests for the data underlying this article should be directed to the administrators of the InterMutualistic Agency (IMA) database or Minimal Hospital Dataset and are subject to approval.
