# Supplementary material for: Anticoagulant-related bleeding as a sign of underlying tumoural lesions in patients with atrial fibrillation: a nationwide cohort study
Source: Eur Heart J Open. 2024 Sep 24;4(5):oeae081. doi: 10.1093/ehjopen/oeae081 (PMC11467691; doi:10.1093/ehjopen/oeae081)
Supplement: oeae081_Supplementary_Data [file oeae081_supplementary_data.docx]

**Supplementary materials**

Table of Contents

[Supplemental tables 3](#_Toc168557998)

[**eTable1:** Compliance to the STROBE (Strengthening the Reporting of Observational Studies in Epidemiology) reporting guideline.^1^ 3](#_Toc168557999)

[**eTable2:** Definition of in- and exclusion criteria, comorbidities, medication history and clinical risk scores 5](#_Toc168558000)

[**eTable 3**: Definition of outcomes 22](#_Toc168558001)

## **eTable 4:** A multivariate cause-specific Cox regression on the risk of an incident hematologic neoplasm, among OAC users with versus without a bleeding event 27

[Supplemental figures 28](#_Toc168558002)

[**eFigure1:** Overview of study design 28](#_Toc168558003)

**eFigure 2:** Cumulative incidence function for a diagnosis of a hematologic neoplasm following oral anticoagulant (OAC) initiation in atrial fibrillation patients, stratified according to the experience of a bleeding event. The follow-up started at the onset of an OAC-related bleeding (blue), whereas at the time of OAC initiation in case no bleeding occurred (orange). 29

# Supplemental tables

## **eTable1:** Compliance to the STROBE (Strengthening the Reporting of Observational Studies in Epidemiology) reporting guideline.^1^

|  | **Item No.** | **Recommendation** | **Page No.** |
| --- | --- | --- | --- |
| **Title and abstract** | 1 | (*a*) Indicate the study’s design with a commonly used term in the title or the abstract | 1-3 |
|  |  | (*b*) Provide in the abstract an informative and balanced summary of what was done and what was found | 2-3 |
| **Introduction** | | |  |
| Background/rationale | 2 | Explain the scientific background and rationale for the investigation being reported | 4 |
| Objectives | 3 | State specific objectives, including any prespecified hypotheses | 4 |
| **Methods** | | |  |
| Study design | 4 | Present key elements of study design early in the paper | 5-6 |
| Setting | 5 | Describe the setting, locations, and relevant dates, including periods of recruitment, exposure, follow-up, and data collection | 5-6 |
| Participants | 6 | (*a*) Give the eligibility criteria, and the sources and methods of selection of participants. Describe methods of follow-up | 5-6 |
|  |  | (*b*) For matched studies, give matching criteria and number of exposed and unexposed | 5-6 |
| Variables | 7 | Clearly define all outcomes, exposures, predictors, potential confounders, and effect modifiers. Give diagnostic criteria, if applicable | 6-7, eTable 2-3 |
| Data sources/ measurement | 8* | For each variable of interest, give sources of data and details of methods of assessment (measurement). Describe comparability of assessment methods if there is more than one group | 5-7 |
| Bias | 9 | Describe any efforts to address potential sources of bias | 7-8 |
| Study size | 10 | Explain how the study size was arrived at | 5-6 |
| Quantitative variables | 11 | Explain how quantitative variables were handled in the analyses. If applicable, describe which groupings were chosen and why | 7-8 |
| Statistical methods | 12 | (*a*) Describe all statistical methods, including those used to control for confounding | 7-8 |
|  |  | (*b*) Describe any methods used to examine subgroups and interactions | 7-8 |
|  |  | (*c*) Explain how missing data were addressed | 7-8 |
|  |  | (*d*) If applicable, explain how loss to follow-up was addressed | / |
|  |  | (*e*) Describe any sensitivity analyses | 8 |
| **Results** | | |  |
| Participants | 13* | (a) Report numbers of individuals at each stage of study—eg numbers potentially eligible, examined for eligibility, confirmed eligible, included in the study, completing follow-up, and analysed | 8-9, Table 1, eTable 1 |
|  |  | (b) Give reasons for non-participation at each stage | / |
|  |  | (c) Consider use of a flow diagram | / |
| Descriptive data | 14* | (a) Give characteristics of study participants (eg demographic, clinical, social) and information on exposures and potential confounders | Table 1, eTable 1 |
|  |  | (b) Indicate number of participants with missing data for each variable of interest | Table 1 |
|  |  | (c) Summarise follow-up time (eg, average and total amount) | Table 1, eTable 2 |
| Outcome data | 15* | Report numbers of outcome events or summary measures over time | 9, Table 1, Figure 1-2 |
| Main results | 16 | (*a*) Give unadjusted estimates and, if applicable, confounder-adjusted estimates and their precision (eg, 95% confidence interval). Make clear which confounders were adjusted for and why they were included | 9-15, Table 1-4, Figure 1-2 |
|  |  | (*b*) Report category boundaries when continuous variables were categorized | eTable 2 |
|  |  | (*c*) If relevant, consider translating estimates of relative risk into absolute risk for a meaningful time period | / |
| Other analyses | 17 | Report other analyses done—eg analyses of subgroups and interactions, and sensitivity analyses | 14-15 |
| **Discussion** | | |  |
| Key results | 18 | Summarise key results with reference to study objectives | 15-17 |
| Limitations | 19 | Discuss limitations of the study, taking into account sources of potential bias or imprecision. Discuss both direction and magnitude of any potential bias | 17-18 |
| Interpretation | 20 | Give a cautious overall interpretation of results considering objectives, limitations, multiplicity of analyses, results from similar studies, and other relevant evidence | 15-18 |
| Generalisability | 21 | Discuss the generalisability (external validity) of the study results | 15-18 |
| **Other information** | | |  |
| Funding | 22 | Give the source of funding and the role of the funders for the present study and, if applicable, for the original study on which the present article is based | 19-20 |

**Give information separately for exposed and unexposed groups.*

## **eTable2:** Definition of in- and exclusion criteria, comorbidities, medication history and clinical risk scores

| **Outcome variables** | **ICD, ATC, AND MEDICAL PROCEDURE CODES** |
| --- | --- |
| **Exclusion criteria** |  |
| Recent total hip/knee replacement surgery | **Medical procedure group code:**  N32 (≤6 months before index date) |
| Recent venous thromboembolism  (deep vein thrombosis or pulmonary embolism) | **ICD-9:**  451.1, 451.2, 451.81, 451.89, 451.9, 452, 453.2, 453.3, 453.4, 453.5, 453.77, 453.79, 453.87, 453.89, 671.3, 671.4 (≤6 months before index date)  **ICD-10:**  I80.1, I80.2, I80.3, I80.8, I80.9, I81, I82.2, I82.3, I82.4, I82.5, I82.89, I82.9, O22.3, O22.5, O87.1, O87.3 (≤6 months before index date) |
| Valvular atrial fibrillation |  |
| Moderate-severe mitral stenosis | **ICD-9:**  394.0, 394.2, 396.0, 396.1, 746.5  **ICD-10:**  I05.0, I05.2, I34.2, Q23.2 |
| Mechanical prosthetic heart valve | **ICD-9:**  V43.3  **ICD-10:**  Z95.2  **Medical procedure code:**  159110, 159121, 159132, 159143, 159154, 159165 |
| End-stage renal disease |  |
| CKD stage V (without dialysis) | **ICD-9:**  403.01, 403.11, 403.91, 404.02, 404.12, 404.92, 585.5, 585.6, 586  **ICD-10:**  N18.5, N18.6, N19, I12.0, I13.11 |
| Dialysis | **ICD-9:**  V45.11, V56  **ICD-10:**  Z49, Z99.2  **Medical procedure group code:**  N81 |
| Tumoral lesion | **ICD-9:**  140-209, 223, 230-239, 258.0, V58.0, V58.11, V58.12  **ICD-10:**  C00-C96, D00-D09, D37-D49, E31.2, Z51.0, Z51.11, Z51.12 ATC: L01  **Medical procedure code:**  154873, 154884, 154895, 154906, 157231, 157242, 201191, 201202, 201213, 201224, 220275, 220286, 220371, 220382, 201213, 201224, 226914, 226925, 226936, 226940, 227216, 227220, 227275, 227286, 227636, 227640, 227651, 227662, 227673, 227684, 227695, 227706, 227710, 227721, 227732, 227743, 11 227754, 227765, 227776, 227780, 227791, 227802, 227813, 227824, 227835, 227846, 228012, 228023, 228174, 228185, 228233, 228244, 228255, 228266, 228270, 228281, 228292, 228303, 228314, 228325, 228336, 228340, 230473, 230484, 231033, 231044, 241231, 241242, 241415, 241426, 241430, 241441, 241452, 241463, 241555, 241566, 242012, 242023, 242034, 242045, 242292, 242303, 242314, 242325, 242830, 242841, 242852, 242863, 242874, 242885, 242896, 242900, 243051, 243062, 243073, 243084, 243235, 243246, 243736, 243740, 243751, 243762, 243773, 243784, 244016, 244020, 244031, 244042, 244075, 244086, 244790, 244801, 244856, 244860, 244893, 244904, 244915, 244926, 244930, 244941, 244952, 244963, 244974, 244985, 245512, 245523, 245534, 245545, 246050, 246061, 246072, 246083, 247111, 247122, 247133, 247144, 251753, 251764, 251775, 251786, 254892, 254903, 256115, 256126, 256336, 256340, 256572, 256583, 257191, 257202, 258355, 258366, 258370, 258381, 258392, 258403, 258451, 258462, 258554, 258565, 258856, 258860, 258871, 258882, 258893, 258904, 259033, 259044, 259114, 259125, 260190, 260201, 260411, 260422, 260433, 260444, 260551, 260562, 260654, 260665, 260750, 260761, 261111, 261122, 261391, 261402, 261472, 261483, 261671, 261682, 261774, 261785, 261796, 261800, 262334, 262345, 262570, 262581, 277756, 277760, 277771, 277782, 278795, 278806, 278810, 278821, 281831, 281842, 281956, 281960, 282310, 282321, 282671, 282682, 284056, 284060, 288455, 288466, 288470, 288481, 289892, 289903, 291056, 291060, 310494, 310505, 311312, 311323, 312550, 312561, 312572, 312583, 312594, 312605, 312653, 312664, 312970, 312981, 350114, 350125, 350136, 350140, 350276, 350280, 350291, 350302, 350372, 350383, 350674, 350685, 350696, 350700, 431174, 431185, 431336, 431340, 431351, 431362, 432294, 432305, 444113, 444124, 444135, 444146, 444150, 444161, 444172, 444183, 444194, 444205, 444216, 444220, 444231, 444242, 444253, 444264, 444275, 444286, 444290, 444301, 444312, 444323, 444334, 444345, 444474, 444485, 444592, 444603, 473970, 473981, 474795, 474806, 565073, 565084, 565095, 565106, 565110, 565121, 565132, 565143, 565154, 565165, 587834, 587845, 587871, 587882, 587893, 587904, 587915, 587926, 588431, 588442, 588453, 588464, 588475, 588486, 588490, 588501, 588512, 588523, 588534, 588545, 588556, 588560, 588571, 588582, 588593, 588604, 588770, 588781, 588976, 588980, 589691, 589702, 589713, 589724, 589831, 589842, 589875, 589886, 594016, 594020, 594031, 594042, 594053, 594064, 594075, 594086, 594090, 594101, 594112, 594123, 594252, 594263, 594274, 594285, 594296, 594300, 594311, 594322, 594333, 594344, 594355, 594366, 594370, 594381, 594392, 594403, 594414, 594425, 594436, 594440, 594451, 594462, 594495, 594506, 594510, 594521, 594532, 594543, 594554, 594565, 594576, 594580, 594591, 594602, 594613, 594624, 594635, 594646, 594694, 594705, 594716, 594720, 594753, 594764, 594775, 594786, 594790, 594801, 594812, 594823, 594834, 594845, 594856, 594860, 594871, 594882, 594893, 594904, 594915, 594926, 594930, 594941, 598581, 682636, 682640, 682732, 682743, 687934, 687945, 698051, 698062, 698095, 698106, 698390, 698401, 698456, 698460, 698471, 698482, 698493, 698504, 698530, 698541, 745010, 745021, 745032, 745043, 745113, 745124, 745135, 745146, 745150, 745161 |
| **Exposure variables (bleedings)** |  |
| Clinical relevant non major bleeding (CRNMB) | **ICD-9:**  285.1, 287.8, 287.9, 388.69, 459.0, 596.7, 599.7, 602.1, 620.7, 621.4, 623.6, 626.2, 626.5, 626.6, 626.7, 626.8, 626.9, 627.0, 627.1, 729.92, 784.7, 784.8, 786.30, 786.39, 729.92, 958.2  **ICD-10:**  D62, D68.32, D69.8, D69.9, H92.2, J95.01, M79.81, N02, N30.01, N30.11, N30.21, N30.31, N30.41, N30.81, N30.91, N42.1, N83.6, N83.7, N85.7, N89.7, N92.0, N92.1, N92.3, N92.4, N93.0, N93.8, N93.9, N95.0, N99.510, N99.520, N99.530, R31, R04.0, R04.1, R04.2, R04.89, R04.9, R58, T79.2  **Medical procedure code:**  144605, 144620, 144642, 144664, 144686, 254940, 255242, 257445, 431944 |
| Major bleeding (MB) | **ICD 9:**  336.1, 376.32, 372.72, 364.41, 363.6, 363.72, 362.81, 362.43, 379.23, 360.43, 377.42, 423.0, 430, 431, 432.0, 432.1, 432.9, 455.8, 456.0, 456.20, 530.21, 530.7, 530.82, 531.00, 531.20, 531.40, 531.60, 532.00, 532.20, 532.40, 532.60, 533.00, 533.20, 533.40, 533.60, 534.00, 534.20, 534.40, 534.60, 535.01, 535.11, 535.21, 535.31, 535.41, 535.51, 535.61, 535.71, 537.83, 537.84, 562.02, 562.03, 562.12, 562.13, 568.81, 569.3, 569.85, 569.86, 578.0, 578.1, 578.9, 719.1, 852.0, 852.2, 852.4, 853.0  **ICD 10:**  H05.23, H11.3, H21.0, H31.3, H31.41, H35.6, H35.73, H43.1, H44.81, H47.02, I23.0, I31.2, I60, I61, I62.0, I62.1, I62.9, I85.01, I85.11, J94.2, K22.11, K22.6, K22.8, K25.0, K25.2, K25.4, K25.6, K26.0, K26.2, K26.4, K26.6, K27.0, K27.2, K27.4, K27.6, K28.0, K28.2, K28.4, K28.6, K29.01, K29.21, K29.31, K29.41, K29.51, K29.61, K29.71, K29.81, K29.91, K31.811, K31.82, K50.011, K50.111, K50.811, K50.911, K51.011, K51.211, K51.311, K51.411, K51.511, K51.811, K51.911, K55.21, K57.01, K57.11, K57.13, K57.21, K57.31, K57.33, K57.41, K57.51, K57.53, K57.81, K57.91, K57.93, K62.5, K63.81, K64.9, K66.1, K92.0, K92.1, K92.2, K94.01, K94.11, K94.21, K94.31, G95.19, M25.0, S06.340A, S06.341A, S06.342A, S06.343A, S06.344A, S06.345A, S06.346A, S06.347A, S06.348A, S06.349A, S06.350A, S06.351A, S06.352A, S06.353A, S06.354A, S06.355A, S06.356A, S06.357A, S06.358A, S06.359A, S06.360A, S06.361A, S06.362A, S06.363A, S06.364A, S06.365A, S06.366A, S06.367A, S06.368A, S06.369A, S06.4X0A, S06.4X1A, S06.4X2A, S06.4X3A, S06.4X4A, S06.4X5A, S06.4X6A, S06.4X7A, S06.4X8A, S06.4X9A, S06.5X0A, S06.5X1A, S06.5X2A, S06.5X3A, S06.5X4A, S06.5X5A, S06.5X6A, S06.5X7A, S06.5X8A, S06.5X9A, S06.6X0A, S06.6X1A, S06.6X2A, S06.6X3A, S06.6X4A, S06.6X5A, S06.6X6A, S06.6X7A, S06.6X8A, S06.6X9A  **Medical procedure code:**  227441, 230403, 230425, 230440, 243600, 431620, 472124, 473686, 473782  OR Clinically relevant non-major bleeding with blood transfusion or death within 10 days after admission^2^: **Medical procedure code (blood transfusion):** 752124, 752463, 752581 |
| Intracranial bleeding | **ICD-9:**  430, 432.0, 431, 432.1, 432.9, [852.0](http://www.icd9data.com/2015/Volume1/800-999/850-854/852/852.0.htm), 852.2, 852.4, 853.0  **ICD-10:**  I60, I61, I62, S06.340A, S06.341A, S06.342A, S06.343A, S06.344A, S06.345A, S06.346A, S06.347A, S06.348A, S06.349A, S06.350A, S06.351A, S06.352A, S06.353A, S06.354A, S06.355A, S06.356A, S06.357A, S06.358A, S06.359A, S06.360A, S06.361A, S06.362A, S06.363A, S06.364A, S06.365A, S06.366A, S06.367A, S06.368A, S06.369A S06.4X0A, S06.4X1A, S06.4X2A, S06.4X3A, S06.4X4A, S06.4X5A, S06.4X6A, S06.4X7A, S06.4X8A, S06.4X9A S06.5X0A, S06.5X1A, S06.5X2A, S06.5X3A, S06.5X4A, S06.5X5A, S06.5X6A, S06.5X7A, S06.5X8A, S06.5X9A S06.6X0A, S06.6X1A, S06.6X2A, S06.6X3A, S06.6X4A, S06.6X5A, S06.6X6A, S06.6X7A, S06.6X8A, S06.6X9A,  **Medical procedure code:**  230440, 230403, 230425 |
| Gastrointestinal bleeding | **ICD-9:**  456.0, 456.20, 530.21, 530.7, 530.82, 531.0, 531.2, 531.4, 531.6, 532.0, 532.2, 532.40, 532.6, 533.0, 533.2, 533.4, 533.6, 534.0, 534.2, 534.4, 534.6, 535.01, 535.11, 535.21, 535.31, 535.41, 535.51, 535.61, 535.71, 537.83, 537.84, 578.0  **ICD-10:**  I85.01, I85.11, K22.11, K22.6, K22.8, K25.0, K25.2, K25.4, K25.6, K26.0, K26.2, K26.4, K26.6, K27.0, K27.2, K27.4, K27.6, K28.0, K28.2, K28.4, K28.6, K29.01, K29.21, K29.31, K29.41, K29.51, K29.61, K29.71, K29.81, K29.91, K31.811, K31.82, K92.0, K92.1, K94.21, K94.31, K50.011, K50.111, K50.811, K50.911, K51.011, K51.211, K51.311, K51.411, K51.511, K51.811, K51.911, K55.21, K57.01, K57.11, K57.13, K57.21, K57.31, K57.33, K57.41, K57.51, K57.53, K57.81, K57.91, K57.93, K62.5, K63.81, K64.9, K92.2, K94.01, K94.11  **Medical procedure code:**  472124, 473686, 473782 |
| Respiratory tract bleeding | **ICD-9:**  786.3, 784.7, 784.8  **ICD-10:**  R04.2, R04.8, R04.9, J94.1, J94.2  **Medical procedure code:**  227441 |
| Urogenital bleeding | **ICD-9:**  596.7,599.7, 602.1  **ICD-10:**  N02, N30.01, N30.11, N30.21, N30.31, N30.41, N30.81, N30.91, N42.1, N99.510, N99.520, N99.530, R31 |
| **Exposure variables (medication)** |  |
| VKA | **ATC:**  B01AA03, B01AA04, B01AA07 |
| NOAC | **ATC:**  B01AE07, B01AF01, B01AF02, B01AF03 |
| **Comorbidities** |  |
| Anemia | **ICD-9:**  280-285  **ICD-10:**  D46.0-D46.4, D50-D53, D56-D64 |
| Chronic kidney disease | **ICD-9:**  249.4, 250.4, 403.00, 403.10, 403.90, 404.00, 404.10, 404.90, 580, 581, 582, 583, 584, 585.3, 585.4, 585.9, 586, 588, 590.0, 753.12-753.15, 996.81, V42.0  **ICD-10:**  E08.2, E09.2, E10.2, E11.2, E13.2, I12.9, I13.10, M32.14, M32.15, N00, N01, N02, N03, N04, N05, N07, N11, N14, N15.0, N17, N18.3, N18.4, N18.9, N19, N25, Q61.1-Q61.4, T86.1, Z94.0  **Medical procedure code:**  107096, 107111, 107133, 107155, 318010, 318021, 318290, 318301, 754294, 757433, 757492 |
| Chronic liver disease | **ICD-9:**  070.0, 070.2, 070.3, 070.4, 070.51, 070.52, 070.54, 070.6, 070.70, 070.71, 155.0, 155.1, 155.2, 197.7, 456.0, 456.1, 456.2, 567.23, 570, 571, 571.2, 571.5, 571.6, 572.2, 572.3, 572.4, 572.8, 573.0, 573.5, 573.8, 573.9, 789.59, V42.7  **ICD-10:**  B15.0, B16.0, B16.2, B17.0, B17.10, B17.11, B18, B19.0, B19.10, B19.11, B19.20, B19.21, C22, C78.7, I85, I86.4, K65.2, K70.0, K70.1, K70.2, K70.3, K70.4, K70.9, K71.1, K71.3, K71.4, K71.5, K71.6, K71.7, K71.8, K71.9, K72, K73, K74, K75.3, K75. 4, K75.8, K75.9, K76.0, K76.1, K76.2, K76.5, K76.6, K76.7, K76.81, K76.89, K76.9, K77, R18.8, Z94.4  **ATC:**  J05AB04, J05AF05, J05AF07, J05AF08, J05AF10, J05AE11, J05AE12, J05AE14, J05AX15, J05AX65, J05AP  **Medical procedure code:**  318076, 318080, 318334, 318345, 472113, 472124, 556754, 556765, 589352, 589363 |
| Chronic lung disease | **ICD-9:**  416, 491-496, 500-505, 506.4, 508.1, 515, 516.3, 516.9, 518.1, 518.2, 518.83, 518.84  **ICD-10:**  I27, J41-J45, J47, J60-J67, J68.4, J70.1, J70.3, J84.1, J84.9, J96.1, J96.2, J98.2, J98.3  **ATC:** R03DC, R03DX |
| Coronary artery disease | **ICD 9:**  410, 411, 412, 413, 414, 429.2, 429.7, V45.81, V45.82  **ICD 10:**  I20, I21, I22, I23, I24, I25, Z95.1, Z95.5, Z98.61 |
| Diabetes mellitus | **ICD-9:**  249, 250, 357.2, 362.0, 366.41, V45.85, V53.91, V65.46  **ICD-10:**  E8, E9, E10, E11, E13, Z46.81, Z96.41  **ATC:**  A10  **Medical procedure code:**  102852, 107015, 107030, 107052, 107074, 109594, 174370, 174381, 174392, 174403, 174414, 174425, 174436, 174440, 174451, 174462, 174473, 174484, 174495, 174506, 174510, 174521, 653671, 653682, 697093, 697104, 754176, 754191, 754250, 754272, 754736, 757352, 757374, 757396, 757411, 757514, 757536, 757551, 770070, 773393, 773496, 784630, 784641, 784652, 784663, 785735, 785750, 785772, 785794, 785816, 785831, 785853, 785875, 785890, 785912, 785934, 785956, 786015, 786030, 786100, 788756, 788771, 788793, 788815, 788830, 788852, 788874, 788896, 788911, 788933, 788955, 789751, 789773, 789795, 789810, 789832, 789854, 789876, 789891, 789913, 789935, 794032, 794113, 794135, 794150, 794194, 794216, 794231, 794253, 794275, 794290, 794312, 794334, 794356, 794371, 794393, 794415, 794430, 794452, 961295, 961306, 961332, 961343 |
| Dyslipidaemia | **ICD 9:**  272.0, 272.1, 272.3, 272.4, 272.5, 272.8, 272.9  **ICD-10:**  E78  **ATC:**  C10(>90 DPP’s) |
| Hypertension | **ICD-9:**  401-405, 437.2  **ICD-10:**  I10-I13, I15, I16, I67.4  **ATC:** Combination treatment with ≥2 of the following drug classes:   - Cardioselective beta blocker: C07AB, C07AG, C07BB, C07BG, C07CB, C07CG, C07DB, C07FB, C07FX03, C07FX04, C07FX05, C07FX06, C09BX02, C09BX04, C09BX05, C09DX05 - ACE inhibitor or angiotensin II receptor blocker: C09A, C09B, C09C, C09D, C10BX04, C10BX06, C10BX07, C10BX10, C10BX11, C10BX12, C10BX13, C10BX14, C10BX15, C10BX16, C10BX17, C10BX18 - Calcium channel blocker: C07FB, C08C, C08G, C09BB, C09DB, C09BX01, C09BX03, C09BX04, C09DX01, C09DX03, C09DX06, C09DX07, C09XA53, C09XA54, C10BX03, C10BX07, C10BX09, C10BX11, C10BX14, C10BX18 - Non-loop diuretic: C02L, C03A, C03BA, C03BB, C03EA, C07B, C07C, C07D, C08GA, C09BA, C09BX01, C09BX03, C09DA, C09DX01, C09DX03, C09DX06, C09DX07, C09XA52, C09XA54, C10BX13, C03D, C03EA, C03EB - Other antihypertensive (alpha adrenergic blocker, vasodilator): C02A, C02B, C02C, C02DB, C02DD, C02DG, C02L |
| Inflammatory bowel disease (IBD) | Crohn disease   - **ICD-9:** 555 - **ICD-10:** K50   ulcerative colitis   - **ICD-9:** 556 - **ICD-10:** K51 |
| Lower GI disorder | **ICD-9:**  211.3, 211.4, 448.0, 455, 537.82, 537.83, 537.84, 562, 569.84, 569.85, 569.86, V12.72  **ICD-10:**  D12, I78.0, K31.81, K31.82, K55.2, K57, K63.5, K64, K63.81, Z86.010  **Medical procedure code:**  112313, 112324, 243294, 243305, 244311, 244322, 244355, 244366, 244370, 244381, 244554, 244565, 244576, 244580, 244591, 244602, 472150, 472161, 473211, 473222, 473476, 473480, 473675, 473686, 473955, 473966, 473970, 473981, 474795, 474806 |
| Peripheral artery disease | **ICD-9:**  440, 441, 443.89, 443.9, 444, 447.1, 557.1, 557.9, V43.4  **ICD-10:**  I70, I71, I73.8, I73.9, I74, I77.1, I79.0, K55.1, K55.8, K55.9, Z95.82, Z98.62  **Medical procedure code:**  229294, 229305, 229316, 229320, 229331, 229342, 235071, 235082, 235093, 235104, 235115, 235126, 235196, 235200, 235211, 235222, 236014, 236025, 236036, 236040, 236051, 236062, 237016, 237020, 237031, 237042, 237053, 237064, 237075, 237086, 237090, 237101, 237171, 237182, 589050, 589061, 589094, 589105, 589175, 589186, 589595, 589606, 589610, 589621, 589632, 589643, 589654, 589665 |
| Pneumonia | **ICD-9:**  480, 481, 482, 483, 484, 485, 486, 487.0  **ICD-10:**  A48.1, J11.0, J12, J13, J14, J15, J16, J17, J18 |
| Upper GI disorders | **ICD-9:**  041.86, 530.1, 530.2, 530.81, 530.85, 531, 532, 533, 534, 535, V12.71  **ICD-10:**  B96.81, K20, K21, K22.1, K22.7, K25, K26, K27, K28, K29, Z87.11  **ATC:**  A02BD04, A02BD11  **Medical procedure code:**  172616, 172620, 172631, 172642, 172653, 172664, 474854, 474865, 550093, 550104, 552370, 552381 |
| **Medication history** |  |
| Antiplatelet | **ATC:** B01AB, B01AC, B01AX, C07FX02, C07FX03, C07FX04, C10BX01, C10BX02, C10BX04, C10BX05, C10BX06, C10BX08, C10BX12, M01A, N02BA01, N02BA51, N02BA71 |
| NSAID | **ATC:** M01AA, M01AB, M01AC, M01AE, M01AG, M01AH, N02AJ08, N02AJ14, N02AJ19, C08CA51 |
| **Clinical risk scores (at baseline)** |  |
| CHA2DS2-VASc score^3^ | 1. Congestive heart failure: 1 point:  - **ICD-9:** 398.91, 402.01, 402.11, 402.91, 404.01, 404.03, 404.11, 404.13, 404.91, 404.93, 425.4–425.9, 428 - **ICD-10:** I09.81, I11.0, I13.0, I13.2, I42.0, I42.6-I42.9, I43, I50 - **ATC:** combination treatment of the following classes of drugs:   - Cardioselective beta blockers: C07AB, C07AG, C07BB, C07BG, C07CB, C07CG, C07DB, C07FB, C07FX03, C07FX04, C07FX05, C07FX06, C09BX02, C09BX04, C09BX05, C09DX05   - ACE inhibitors or angiotensin II receptor blockers: C09A, C09B, C09C, C09D, C10BX04, C10BX06, C10BX07, C10BX10, C10BX11, C10BX12, C10BX13, C10BX14, C10BX15, C10BX16, C10BX17, C10BX18   - Potassium-sparing diuretics: C03D, C03EA, C03EB   - Loop diuretics: C03C, C03EB  1. Hypertension: 1 point (definition mentioned above: ‘Hypertension’) 2. Diabetes mellitus: 1 point (definition mentioned above: ‘Diabetes mellitus’) 3. Stroke or systemic embolism:  - Stroke:   - **ICD-9:** 433.01, 433.1, 433.21, 433.31, 433.81, 433.91, 434.01, 434.11, 434.91   - **ICD-10:** I63.0, I63.1, I63.2, I63.3, I63.4, I63.5, I63.8, I63.9   - Medical procedure code: 182136, 182140, 182151, 182162, 182173, 182184 - Systemic embolism:   - **ICD-9:** 431, 432.9, 436, 438, 444, 557.0, 593.81, V12.54   - **ICD-10:** D73.5, I61, I62.9, I67.89, I69.1, I69.2, I69.3, I69.8, I69.9, I74, K55.01, K55.02, N28.0, Z86.73   - **Medical procedure code:** 235130, 235141, 237112, 237123, 477724, 477746, 477761, 477783, 589175, 589186  1. Vascular disease: 1 point (definition mentioned above: ‘Coronary artery disease’ & ‘Peripheral artery disease’) 2. Age 65-74 years: 1 point 3. Age ≥75 years: 2 points 4. Sex category (female): 1 point |
| HAS-BLED score^3^ | 1. Hypertension: 1 point (definition mentioned above: ‘Hypertension’) 2. Abnormal renal function: 1 point (definition mentioned above: ‘Chronic kidney disease’) 3. Abnormal liver function: 1 point (definition mentioned above: ‘Chronic liver disease’) 4. Stroke: 1 point:    - **ICD-9:** 433.01, 433.1, 433.21, 433.31, 433.81, 433.91, 434.01, 434.11, 434.91    - **ICD-10:** I63.0, I63.1, I63.2, I63.3, I63.4, I63.5, I63.8, I63.9    - Medical procedure codes: 182136, 182140, 182151, 182162, 182173, 182184 5. Bleeding history or predisposition: 1 point (definition mentioned above: ‘Major or clinically relevant nonmajor bleeding’) 6. Labile INR: not available - Elderly (>65 years or frailty): 1 point 7. Drugs (antiplatelet, NSAID): 1 point (definition mentioned above: ‘NSAID’)    - Antiplatelet:      - **ATC:** B01AB, B01AC, B01AX, C07FX02, C07FX03, C07FX04, C10BX01, C10BX02, C10BX04, C10BX05, C10BX06, C10BX08, C10BX12, M01A, N02BA01, N02BA51, N02BA71 8. Excessive alcohol drinking: 1 point:    - ICD-9: 265.1, 291, 303, 305.0, 357.5, 425.5, 535.3, 571.0-571.3, 980.0, E860.1, V11.3    - ICD-10: E51, F10, G31.2, G62.1, G72.1, I42.6, K29.2, K70, K85.2, K86, O35.4, T51.0, T51.9, Z71.4    - ATC: N07BB    - Medical procedure code: 790090 |
| Charlson Comorbidity Index^4^ | 1. Myocardial infarction: 1 point: 2. **ICD-9**: 410.00, 410.01, 410.10, 410.11, 410.20, 410.21, 410.30, 410.31, 410.40, 410.41, 410.50, 410.51, 410.60, 410.61, 410.70, 410.71, 410.80, 410.81, 410.90, 410.91 3. **ICD-10**: I21, I22 4. Congestive heart failure: 1 point:  - **ICD 9:** 398.91, 402.01, 402.11, 402.91, 404.01, 404.03, 404.11, 404.13, 404.91, 404.93, 425.4–425.9, 428 - **ICD 10:** I09.81, I11.0, I13.0, I13.2, I42.0, I42.6-I42.9, I43, I50 - **ATC:** combination treatment of the following classes of drugs:   - Cardioselective beta blockers: C07AB, C07AG, C07BB, C07BG, C07CB, C07CG, C07DB, C07FB, C07FX03, C07FX04, C07FX05, C07FX06, C09BX02, C09BX04, C09BX05, C09DX05   - ACE inhibitors or angiotensin II receptor blockers: C09A, C09B, C09C, C09D, C10BX04, C10BX06, C10BX07, C10BX10, C10BX11, C10BX12, C10BX13, C10BX14, C10BX15, C10BX16, C10BX17, C10BX18   - Potassium-sparing diuretics: C03D, C03EA, C03EB   - Loop diuretics: C03C, C03EB  1. Peripheral vascular disease: 1 point (definition mentioned above: ‘Peripheral artery disease’) 2. Cerebrovascular disease: 1 point  - **ICD-9**: 362.34, 430, 431, 432, 433, 434, 435, 436, 437, 438 - **ICD-10**: I61, I62, I63, I65, I66, I67, I68, I69, G45, G46, H34.0  1. Dementia: 1 point  - **ICD-9**: 046.11, 046.19, 290.0, 290.1, 290.2, 290.3, 290.4, 291.1, 294, 331.0, 331.11, 331.19, 331.82 12 - **ICD-10**: A81.0, F01, F02, F03, F10.27, F10.97, G30, G31.0, G31.83, G31.85 - **ATC:** N06D  1. Chronic pulmonary disease: 1 point (definition mentioned above: ‘Chronic lung disease’) 2. Connective tissue disease: 1 point:  - **ICD-9**: 136.1, 287.0, 446, 447.5, 447.6, 710, 711.2, 725, 728.5, 729.30 - **ICD-10**: D69.0, M30, M31, M32, M33, M34, M35, M36.0, M36.8  1. Peptic ulcer disease: 1 point:  - **ICD-9**: 041.86, 530.2, 531, 532, 533, 534, V12.71 - **ICD-10**: B96.81, K22.1, K25, K26, K27, K28, Z87.11 - **ATC**: A02BD04, A02BD11 - **Medical procedure code**: 550093, 550104, 552370, 552381  1. Mild liver disease: 1 point:  - **ICD-9**: 070.3, 070.51, 070.52, 070.54, 070.70, 571, 572.8, 573.0, 573.8, 573.9 - **ICD-10**: B17.0, B17.10, B18, B19.10, B19.20, K70.0, K70.1, K70.2, K70.3, K70.9, K71.3, K71.4, K71.5, K71.6, K71.7, K71.8, K71.9, K73, K74, K75.3, K75.4, K75.8, K75.9, K76.0, K76.1, K76.2, K76.89, K76.9, K77 - **ATC**: J05AB04, J05AF05, J05AF07, J05AF08, J05AF10, J05AE11, J05AE12, J05AE14, J05AX15, J05AX65, J05AP - **Medical procedure code**: 556754, 556765  1. Diabetes without chronic complications: 1 point  - **ICD-9**: 249.4-249.7, 249.9, 250.4-250.7, 250.9, 357.2, 362.0, 364.41 - **ICD-10**: E08.2-E08.5, E08.8, E09.2-E09.5, E09.8, E10.2-E10.5, E10.8, E11.2-E11.5, E11.8, E13.2-E13.5, E13.8 - **Medical procedure code**: 653671, 653682, 697093, 697104, 770070, 773393, 773496  1. Diabetes with chronic complications: 2 points  - **ICD-9**: 249.0-249.3, 249.8, 250.0-250.3, 250.8 - **ICD-10**: E08.0, E08.1, E08.6, E08.9, E09.0, E09.1, E09.6, E09.9, E10.1, E10.6, E10.9, E11.0, E11.1, E11.6, E11.9, E13.0, E13.1, E13.6, E13.9  1. Hemiplegia or paraplegia: 2 points:  - **ICD-9**: 334.1, 342, 343.0, 343.1, 343.2, 343.4, 344.0, 344.1, 344.2, 344.9, 438.2 14 - **ICD-10**: G04.1, G11.4, G80.0, G80.1, G80.2, G81, G82, G83.0, G86.9, I69.05, I69.15, I69.25, I69.35, I69.85, I69.95 - **Medical procedure code:** 643414, 643425  1. Renal disease: 2 points  - **ICD-9:** 403.01, 403.11, 403.91, 404.02, 404.12, 404.92, 585.5, 585.6, 586, 996.81, V42.0, V45.11, V56 - **ICD-10**: N18.5, N18.6, N19, I12.0, I13.11, T86.1, Z49, Z94.0, Z99.2 - **Medical procedure code**: N81 (group code), 318010, 318021, 318290, 318301  1. Any malignancy, including leukemia and lymphoma: 2 points:  - **ICD-9**: 140-195, 199.1, 199.2, 200-209, 223, 230-239, 258.0, V58.0, V58.11, V58.12 - **ICD-10**: C00-C76, C80.1, C80.2, C81-C96, D00-D09, D37-D49, E31.2, Z51.0, Z51.11, Z51.12 - **ATC**: L01 - **Medical procedure code**: 154873, 154884, 154895, 154906, 157231, 157242, 201191, 201202, 201213, 201224, 220275, 220286, 220371, 220382, 201213, 201224, 226914, 226925, 226936, 226940, 227216, 227220, 227275, 227286, 227636, 227640, 227651, 227662, 227673, 227684, 227695, 227706, 227710, 227721, 227732, 227743, 227754, 227765, 227776, 227780, 227791, 227802, 227813, 227824, 227835, 227846, 228012, 228023, 228174, 228185, 228233, 228244, 228255, 228266, 228270, 228281, 228292, 228303, 228314, 228325, 228336, 228340, 230473, 230484, 231033, 231044, 241231, 241242, 241415, 241426, 241430, 241441, 241452, 241463, 241555, 241566, 242012, 242023, 242034, 242045, 242292, 242303, 242314, 242325, 242830, 242841, 242852, 242863, 242874, 242885, 242896, 242900, 243051, 243062, 243073, 243084, 243235, 243246, 243736, 243740, 243751, 243762, 243773, 243784, 244016, 244020, 244031, 244042, 244075, 244086, 244790, 244801, 244856, 244860, 244893, 244904, 244915, 244926, 244930, 244941, 244952, 244963, 244974, 244985, 245512, 245523, 245534, 245545, 246050, 246061, 246072, 246083, 247111, 247122, 247133, 247144, 251753, 251764, 251775, 251786, 254892, 254903, 256115, 256126, 256336, 256340, 256572, 256583, 257191, 257202, 258355, 258366, 258370, 258381, 258392, 258403, 258451, 258462, 258554, 258565, 258856, 258860, 258871, 258882, 258893, 258904, 259033, 259044, 259114, 259125, 260190, 260201, 260411, 260422, 260433, 260444, 260551, 260562, 260654, 260665, 260750, 260761, 261111, 261122, 261391, 261402, 261472, 261483, 261671, 261682, 261774, 261785, 261796, 261800, 262334, 262345, 262570, 262581, 277756, 277760, 277771, 277782, 278795, 278806, 278810, 278821, 281831, 281842, 281956, 281960, 282310, 282321, 282671, 282682, 284056, 284060, 288455, 288466, 288470, 288481, 289892, 289903, 291056, 291060, 310494, 310505, 311312, 311323, 312550, 312561, 312572, 312583, 312594, 312605, 312653, 312664, 312970, 312981, 350114, 350125, 350136, 350140, 350276, 350280, 350291, 350302, 350372, 350383, 350674, 350685, 350696, 350700, 431174, 431185, 431336, 431340, 431351, 431362, 432294, 432305, 444113, 444124, 444135, 444146, 444150, 444161, 444172, 444183, 444194, 444205, 444216, 444220, 444231, 444242, 444253, 444264, 444275, 444286, 444290, 444301, 444312, 444323, 444334, 444345, 444474, 444485, 444592, 444603, 473970, 473981, 474795, 474806, 565073, 565084, 565095, 565106, 565110, 565121, 565132, 565143, 565154, 565165, 587834, 587845, 587871, 587882, 587893, 587904, 587915, 587926, 588431, 588442, 588453, 588464, 588475, 588486, 588490, 588501, 588512, 588523, 588534, 588545, 588556, 588560, 588571, 588582, 588593, 588604, 588770, 588781, 588976, 588980, 589691, 589702, 589713, 589724, 589831, 589842, 589875, 589886, 594016, 594020, 594031, 594042, 594053, 594064, 594075, 594086, 594090, 594101, 594112, 594123, 594252, 594263, 594274, 594285, 594296, 594300, 594311, 594322, 594333, 594344, 594355, 594366, 594370, 594381, 594392, 594403, 594414, 594425, 594436, 594440, 594451, 594462, 594495, 594506, 594510, 594521, 594532, 594543, 594554, 594565, 594576, 594580, 594591, 594602, 594613, 594624, 594635, 594646, 594694, 594705, 594716, 594720, 594753, 594764, 594775, 594786, 594790, 594801, 594812, 594823, 594834, 594845, 594856, 594860, 594871, 594882, 594893, 594904, 594915, 594926, 594930, 594941, 598581, 682636, 682640, 682732, 682743, 687934, 687945, 698051, 698062, 698095, 698106, 698390, 698401, 698456, 698460, 698471, 698482, 698493, 698504, 698530, 698541, 745010, 745021, 745032, 745043, 745113, 745124, 745135, 745146, 745150, 745161  1. Moderate or severe liver disease: 3 points  - **ICD-9**: 070.0, 070.2, 070.4, 070.6, 070.71, 155.0, 155.1, 155.2, 197.7, 456.0, 456.1, 456.2, 567.23, 570, 571.2, 571.5, 571.6, 572.2, 572.3, 572.4, 573.0, 573.5, 789.59, V42.7 - **ICD-10**: B15.0, B16.0, B16.2, B17.11, B19.0, B19.11, B19.21, C22, C78.7, I85, I86.4, K65.2, K70.2, K70.3, K70.4, K71.1, K71.7, K72, K74, K76.1, K76.5, K76.6, K76.7, K76.81, R18.8, Z94.4 - **Medical procedure code**: 318076, 318080, 318334, 318345, 472113, 472124, 589352, 589363  1. Metastatic solid tumor: 6 points  - **ICD-9**: 196-198, 199.0 - **ICD-10**: C77-C79, C80.0  1. AIDS/HIV: 6 points  - **ICD-9**: 042, V08 - **ICD-10**: B20, Z21 - ATC: J05AE01, J05AE02, J05AE03, J05AE04, J05AE05, J05AE07, J05AE08, J05AE09, J05AE10, J05AF01, J05AF02, J05AF03, J05AF04, J05AF05, J05AF06, J05AF09, J05AF11, J05AF12, J05AF13, J05AG, J05AR, J05AX07, J05AX08, J05AX09, J05AX12  1. Age:   <50 years: 0 points  50-59 years: 1 point  60-69 years: 2 points  70-79 years: 3 points  ≥80 years: 4 points  *The following comorbid conditions were mutually exclusive: diabetes with chronic complications and diabetes without chronic complications; mild liver disease and moderate or severe liver disease; and any malignancy and metastatic solid tumor.* |
| John Hopkins Claims-based Frailty Indicator^5^ | 1. **Impaired mobility**: beta coefficient 1.24:    - **ICD-9:** 334, 719.7, 781.2, V46.3, V49.84, V57.81    - **ICD-10:** G11, G32.81, M62.3, R26, R29.6, Z74.01, Z74.09, Z99.3    - **Medical procedure code:** N83, 643451, 643462, 653656, 653660, 770394, 770405, 770416, 770420 2. **Depression**: beta coefficient 0.54:    - **ICD-9**: 293.83, 296, 300.4, 301.12, 309.0, 309.1, 309.28, 311    - **ICD-10**: F06.31, F06.32, F30, F31, F32, F33, F34.1, F43.21, F43.23    - **ATC:** N06A 3. **Congestive heart failure**: beta coefficient 0.50: (definition mentioned above: ‘Congestive heart failure’) 4. **Parkinson’s disease**: beta coefficient 0.50: (definition mentioned above: ‘Parkinson’s disease’) 5. **White race**: beta coefficient -0.49: not available 6. **Arthritis (any type):** beta coefficient 0.43:    - **ICD-9:** 099.3, 696.0, 711.1, 711.3, 713.1, 714.0, 714.1, 714.2, 714.3, 714.4, 714.8, 714.9, 716.5, 716.6, 720.0, 720.2, 720.89, 720.9, V13.4    - **ICD-10:** L40.5, M02.1, M02.3, M05, M06, M07, M08, M13.0, M13.1, M45, M46.1, M46.8, M46.9, Z87.39    - **ATC:** L04AA13, L04AA24, L04AA29, L04AA37    - **Medical procedure code:** 478030, 478041 7. **Cognitive impairment**: beta coefficient 0.33:    - **ICD-9:** 331.2, 331.83, 331.89, 331.9, 797    - **ICD-10:** G31.1, G31.84, G31.89, G31.9, R41.81 8. **Charlson comorbidity index** **(> 0)**: beta coefficient 0.31 9. **Stroke**: beta coefficient 0.28: (definition mentioned above: ‘Stroke’) 10. **Paranoia**: beta coefficient 0.24:     - **ICD-9:** 293.81, 293.82, 295, 297, 298     - **ICD-10:** F06.0, F06.2, F20, F22, F23, F24, F28, F29 11. **Chronic skin ulcer**: beta coefficient 0.23:     - **ICD-9:** 707     - **ICD-10:** E08.621, E08.622, E09.621, E09.622, E10.621, E10.622, E11.621, E11.622, E13.621, E13.622, L89, L97, L98.4     - **Medical procedure code:** 114074, 114085 12. **Pneumonia**: beta coefficient 0.21:     - **ICD-9:** 480, 481, 482, 483, 484, 485, 486, 487.0     - **ICD-10:** A48.1, J11.0, J12, J13, J14, J15, J16, J17, J18 13. **Male sex**: beta coefficient -0.19 14. **Skin and soft tissue infection**: beta coefficient 0.18:     - **ICD-9:** 680, 681, 682, 683, 684, 685, 686, 695.81     - **ICD-10:** L00, L01, L02, L03, L04, L05, L08 15. **Mycoses**: beta coefficient 0.14:     - **ICD-9:** 110, 111, 112, 114, 115, 116, 117, 118     - **ICD-10:** B35, B36, B37, B38, B39, B40, B41, B42, B43, B44, B45, B46, B47, B48, B49 16. **Age (in 5 year categories)**: beta coefficient 0.09 17. **Admission in past 6 months**: beta coefficient 0.09 18. **Gout or other crystal-induced arthropathy**: beta coefficient 0.08:     - **ICD-9:** 274, 712.3, 712.8, 712.90     - **ICD-10:** M10, M11, M1A     - **ATC:** M04A 19. **Falls**: beta coefficient 0.08: (definition mentioned above: ‘History of falls’) 20. **Musculoskeletal problems**: beta coefficient 0.05:     - **ICD-9:** 713, 716.0, 716.2, 716.3, 716.4, 716.5, 716.6, 716.8, 716.9, 718.1, 718.2, 718.5, 718.6, 718.7, 718.8, 718.9, 719-724, 733.0, 733.1, 733.93, 733.94, 733.95, 733.96, 733.97, 733.98, V13.51, V13.52     - **ICD-10:** M07, M12.0, M12.1, M12.2, M12.3, M12.4, M12.8, M12.9, M13, M14, M24.0, M24.3, M24.6, M24.7, M24.8, M24.9, M25, M45, M46.0, M46.1, M46.4, M46.8, M46.9, M47-M51, M53, M54, M80, M81, M84.3, M84.4, M84.5, M84.6, Z87.31 21. **Urinary tract infection**: beta coefficient 0.05:     - **ICD-9:** 590.1, 590.8, 590.9, 595.0, 595.4, 595.89, 595.9, 597, 599.0     - **ICD-10:** N30.0, N30.8, N30.9, N10, N12, N13.6, N15.9, N16, N34, N39.0     - **ATC:** J01XE01, J01XX01 |

*Definition of in- and exclusion criteria, comorbidities, medication history and clinical risk scores based on ICD-coded hospital discharge diagnoses (ICD-9-CM up to 2014 and ICD-10-BE from 2015 onward)^6^, medical procedure codes^7^ and/or ATC-coded prescription claims^8^.*

*AF: Atrial fibrillation; ATC: Anatomical Therapeutic Chemical Classification; CKD: Chronic kidney disease; ICD-9-CM: International Classification of Diseases (ICD) codes, 9th revision, Clinical Modification; ICD-10-BE: International Classification of Diseases (ICD) codes, 10th Revision, Belgian Modification; INR: International Normalized Ratio; NOAC: non-vitamin K antagonist oral anticoagulant; NSAID: non-steroidal anti-inflammatory drug; OAC: oral anticoagulant; VKA: vitamin K antagonist; y: year.*

## **eTable 3**: Definition of outcomes

| **Outcome variables** | **ICD, ATC, AND MEDICAL PROCEDURE CODES** |
| --- | --- |
| Tumoral lesion | **ICD-9:**  140-209, 223, 230-239, 258.0, V58.0, V58.11, V58.12  **ICD-10:**  C00-C96, D00-D09, D37-D49, E31.2, Z51.0, Z51.11, Z51.12 ATC: L01  **Medical procedure code:**  154873, 154884, 154895, 154906, 157231, 157242, 201191, 201202, 201213, 201224, 220275, 220286, 220371, 220382, 201213, 201224, 226914, 226925, 226936, 226940, 227216, 227220, 227275, 227286, 227636, 227640, 227651, 227662, 227673, 227684, 227695, 227706, 227710, 227721, 227732, 227743, 11 227754, 227765, 227776, 227780, 227791, 227802, 227813, 227824, 227835, 227846, 228012, 228023, 228174, 228185, 228233, 228244, 228255, 228266, 228270, 228281, 228292, 228303, 228314, 228325, 228336, 228340, 230473, 230484, 231033, 231044, 241231, 241242, 241415, 241426, 241430, 241441, 241452, 241463, 241555, 241566, 242012, 242023, 242034, 242045, 242292, 242303, 242314, 242325, 242830, 242841, 242852, 242863, 242874, 242885, 242896, 242900, 243051, 243062, 243073, 243084, 243235, 243246, 243736, 243740, 243751, 243762, 243773, 243784, 244016, 244020, 244031, 244042, 244075, 244086, 244790, 244801, 244856, 244860, 244893, 244904, 244915, 244926, 244930, 244941, 244952, 244963, 244974, 244985, 245512, 245523, 245534, 245545, 246050, 246061, 246072, 246083, 247111, 247122, 247133, 247144, 251753, 251764, 251775, 251786, 254892, 254903, 256115, 256126, 256336, 256340, 256572, 256583, 257191, 257202, 258355, 258366, 258370, 258381, 258392, 258403, 258451, 258462, 258554, 258565, 258856, 258860, 258871, 258882, 258893, 258904, 259033, 259044, 259114, 259125, 260190, 260201, 260411, 260422, 260433, 260444, 260551, 260562, 260654, 260665, 260750, 260761, 261111, 261122, 261391, 261402, 261472, 261483, 261671, 261682, 261774, 261785, 261796, 261800, 262334, 262345, 262570, 262581, 277756, 277760, 277771, 277782, 278795, 278806, 278810, 278821, 281831, 281842, 281956, 281960, 282310, 282321, 282671, 282682, 284056, 284060, 288455, 288466, 288470, 288481, 289892, 289903, 291056, 291060, 310494, 310505, 311312, 311323, 312550, 312561, 312572, 312583, 312594, 312605, 312653, 312664, 312970, 312981, 350114, 350125, 350136, 350140, 350276, 350280, 350291, 350302, 350372, 350383, 350674, 350685, 350696, 350700, 431174, 431185, 431336, 431340, 431351, 431362, 432294, 432305, 444113, 444124, 444135, 444146, 444150, 444161, 444172, 444183, 444194, 444205, 444216, 444220, 444231, 444242, 444253, 444264, 444275, 444286, 444290, 444301, 444312, 444323, 444334, 444345, 444474, 444485, 444592, 444603, 473970, 473981, 474795, 474806, 565073, 565084, 565095, 565106, 565110, 565121, 565132, 565143, 565154, 565165, 587834, 587845, 587871, 587882, 587893, 587904, 587915, 587926, 588431, 588442, 588453, 588464, 588475, 588486, 588490, 588501, 588512, 588523, 588534, 588545, 588556, 588560, 588571, 588582, 588593, 588604, 588770, 588781, 588976, 588980, 589691, 589702, 589713, 589724, 589831, 589842, 589875, 589886, 594016, 594020, 594031, 594042, 594053, 594064, 594075, 594086, 594090, 594101, 594112, 594123, 594252, 594263, 594274, 594285, 594296, 594300, 594311, 594322, 594333, 594344, 594355, 594366, 594370, 594381, 594392, 594403, 594414, 594425, 594436, 594440, 594451, 594462, 594495, 594506, 594510, 594521, 594532, 594543, 594554, 594565, 594576, 594580, 594591, 594602, 594613, 594624, 594635, 594646, 594694, 594705, 594716, 594720, 594753, 594764, 594775, 594786, 594790, 594801, 594812, 594823, 594834, 594845, 594856, 594860, 594871, 594882, 594893, 594904, 594915, 594926, 594930, 594941, 598581, 682636, 682640, 682732, 682743, 687934, 687945, 698051, 698062, 698095, 698106, 698390, 698401, 698456, 698460, 698471, 698482, 698493, 698504, 698530, 698541, 745010, 745021, 745032, 745043, 745113, 745124, 745135, 745146, 745150, 745161 |
| Intracranial tumoral lesion | **ICD-9:**  191, 192.1, 225, 2375, 2396, C70, C71, C72.5, D43.0, D43.1, D43.2, D43.3, D49.6  **ICD-10:**  C70.0, C71, C79.3, D43.0, D43.1, D43.2, D49.6  **Medical procedure code:**  230473, 230484, 231033, 231044, 284056, 284060 |
| Gastrointestinal tumoral lesion | **ICD-9:**  150, 151, 152, 153, 154, 209, 211, 230,  **ICD-10:**  C15, C16, C17, C18, C19, C20, C21, C26.0, C26.9, C78.4, C78.5, C7A, C7B, D00.1, D00.2, D01.0, D01.1, D01.2, D01.3, D01.4, D01.9, D37.1, D37.2, D37.3, D37.4, D37.5, D37.9, D49.0  **Medical procedure code:**  228012, 228023, 228174, 228185, 228233, 228244, 228255, 228266, 228270, 228281, 228292, 228303, 228314, 228325, 228336, 228340, 241415, 241426, 241430, 241441, 241452, 241463, 241555, 241566, 243051, 243062, 243073, 243084, 243235, 243246, 244016, 244020, 244031, 244042, 244075 244086 244790 244801 473970, 473981 474795 474806, 350674 350685 350696 350700 594274 594285 594252 594263 |
| Respiratory tract tumoral lesion | - **Upper respiratory tract**   **ICD-9:**  146, 147, 148, 161, 212.1  **ICD-10:**  C10, C11, C12, C13, C32  **Medical procedure code:**  256572, 256583, 258904, 687934, 687945, 257191, 257202, 258856, 258860, 258871, 258882   - **Lower respiratory tract**   **ICD-9:**  162, 212.2, 212.3  **ICD-10:**  C33, C34, D381  **Medical procedure code:**  227216, 227220, 227275, 227286, 594311, 594322, 594333, 594344, 594355, 594366, 594370, 594381   - **Other**   **ICD-9:**  163, 165, 212.4, 212.8, 212.9, 231  **ICD-10:**  C38.4, C39, C78.0, C78.2, D38.2, D49.1  **Medical procedure code:**  259033, 259044, 259114, 259125, |
| Urological tumoral lesion | - **Bladder**   **ICD-9:**  188, 233.7, 236.7, 239.4  **ICD-10:**  C67, D09.0, D49.4  **Medical procedure code:**  260433, 260444, 260551, 260562, 261391, 261402   - **Prostate**   **ICD-9:**  185, 233.5, 236.5  **ICD-10:**  C61, D07.4, D07.5, D40.0  **Medical procedure code:**  154873, 154884, 154895, 154906, 682636, 682640 682732, 682743, 261796, 261800   - **Other**   **ICD-9:**  189, 198.0, 198.1,223, 233.9, 236.9, 223  **ICD-10:**  C64, C65, C66, C68, C79.0, D09.1, D41, D49.5  **Medical procedure code:**  260190, 260201, 260411, 260422, 260750, 260761, 261774, 261785, 262334, 262345 |
| Hematological cancer | **ICD-9:**  196,200,201,202,203,204,205,206,207,208,228  **ICD-10:**  C81,C82,C83,C84,C85,C86,C88,C90,C91,C92,  C93,C94,C95,C96,D45,D46,D47  **Medical procedure code:**  587834,587845,587871,587882,587893,587904,588431,  588442,588453,588464,588475,588486,588490,588501,  588512,588523,588571,588582,588593,588604,588770,  588781,594495,594506,594510,594521,594532,594543,  594554,594565,594576,594580,594591,594602,594613,  594624,594635,594646,594694,594705,594716,594720,  594753,594764,594775,594786,594790,594801,594812,  594823,594834,594845,594856,594860,594871,594882,  594893,594904,594915,594926,594930,594941 |

*Definition of the outcome of interest, based on ICD-coded hospital discharge diagnoses (ICD-9-CM up to 2014 and ICD-10-BE from 2015 onward)^6^, medical procedure codes^7^ and/or ATC-coded prescription claims^8^.*

*ATC: Anatomical Therapeutic Chemical Classification; ICD-9-CM: International Classification of Diseases (ICD) codes, 9th revision, Clinical Modification; ICD-10-BE: International Classification of Diseases (ICD) codes, 10th Revision, Belgian Modification; INR: International Normalized Ratio;*

## **eTable 4:** A multivariate cause-specific Cox regression on the risk of an incident hematologic neoplasm, among OAC users with versus without a bleeding event.

| Variables | Hematologic neoplasm aHR (95%CI)cancer HR (95%CI) |
| --- | --- |
| Bleeding effect (MB/CRNMB) | |
| Bleeding after OAC initiation | 2.99(2.60-3.43) |
| Type OAC related to bleeding (ref=VKA) | |
| NOAC related bleeding | 1.06(0.91-1.24) |
| Demographics | |
| Age | 1.03(1.03-1.03) |
| Sex | 0.71(0.66-0.76) |
| Comorbidities | |
| Hypertension | 0.93(0.85-1.01) |
| CAD | 0.84(0.77-0.92) |
| Peripheral artery disease | 1.04(0.92-1.19) |
| Dyslipidemia | 0.88(0.83-0.93) |
| Chronic kidney disease | 1.25(1.12-1.39) |
| Chronic liver disease | 1.30(1.07-1.57) |
| Chronic lung disease | 1.12(1.03-1.22) |
| Pneumonia | 1.10(0.96-1.26) |
| Upper GI tract disorder* | 1.04(0.93-1.16) |
| Lower GI tract disorder[**](javascript:;) | 0.95(0.85-1.08) |
| Inflammatory bowel disease | 1.66(1.21-2.29) |
| Diabetes mellitus | 0.97(0.90-1.05) |
| Anemia | 1.38(1.20-1.59) |
| Comorbidity scores | |
| CCI | 0.98(0.94-1.01) |
| CHA2DS2-VASc score | 1.06(1.01-1.10) |
| Frailty | 0.71(0.54-0.92) |
| HAS-BLED score | 0.95(0.90-1.00) |
| Medication usage | |
| Drug number at baseline | 1.03(1.02-1.04) |
| NSAID | 1.04(0.97-1.11) |

**Upper gastrointestinal tract disorders were defined as gastroesophageal reflux diseases or peptic ulcer disease. **Lower gastrointestinal tract disorder was defined as diverticulosis, angiodysplasia, colorectal polyposis or hemorrhoids. aHR: adjusted hazard ratio; CCI: Charlson comorbidity index; CI: confidence interval; CRNMB: clinically relevant non-major bleeding; GI: Gastrointestinal, MB: Major bleeding NOAC: non-vitamin K antagonist oral anticoagulant;*[*NSAID*](https://www.sciencedirect.com/topics/medicine-and-dentistry/nonsteroid-antiinflammatory-agent)*: non-steroidal anti-inflammatory drug; OAC: oral anticoagulant; SE: systemic embolism; sd: standard deviation; VKA: vitamin K antagonist.*

# Supplemental figures

## **eFigure1:** Overview of study design

**
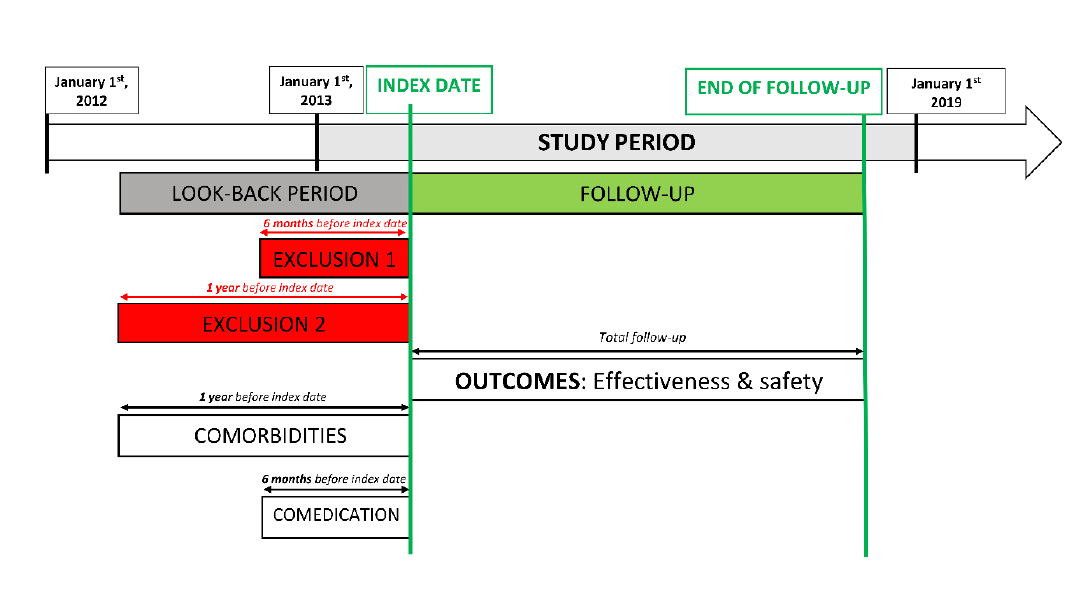
**

***Study period:*** *January 1st, 2013 – January 1st, 2019.* ***Look-back period:*** *One year look back period from the index date (maximum up until January 1st, 2012).* ***Index date:*** *Onset of oral anticoagulant usage (NOAC or VKA) during the study period for subjects ≥45 years old.* ***End of follow-up:*** *Patients were followed from the index date (OAC initiation) till the first occurrence of the investigated outcome, an incident diagnosis of a tumoral lesion, death, emigration, or end of the study period (January 1st, 2019).* ***Exclusion criteria:*** *(1) subjects were OAC naïve, therefore people with an OAC prescription within one year before the index date, were excluded. (2) A total knee or hip replacement, a deep vein thrombosis, or pulmonary embolism less than 6 months before the index date, (3) valvular atrial fibrillation (moderate or severe mitral stenosis or a mechanical prosthetic heart valve), (4) an end-stage renal disease. (5) At index, more than one prescription claim of different OAC doses or types, (6) not approved NOAC doses for stroke prevention in atrial fibrillation. (7) A diagnosis of a tumoral lesion within one year before index date.* ***Comorbidities:*** *Comorbidities were identified using specific ICD-coded diagnoses (e.g. tumoral lesion) from the MHD, medical procedure codes (e.g. cancer-related surgery) from the IMA database and/or ATC-coded prescription claims (e.g. antineoplastic drugs) from the IMA database ≤1 year before the index date.* ***Comedication use:*** *Comedication dispensed up to 6 months before the index date.*

*AF: Atrial fibrillation; ATC: Anatomical Therapeutic Chemical Classification; IMA: InterMutualistic Agency; MHD: Minimal Hospital Dataset; NOAC: non-vitamin K antagonist oral anticoagulant; OAC: oral anticoagulant; VKA: vitamin K antagonist.*

**eFigure 2:** Cumulative incidence function for a diagnosis of a hematologic neoplasm following oral anticoagulant (OAC) initiation in atrial fibrillation patients, stratified according to the experience of a bleeding event. The follow-up started at the onset of an OAC-related bleeding (blue), whereas at the time of OAC initiation in case no bleeding occurred (orange).

**
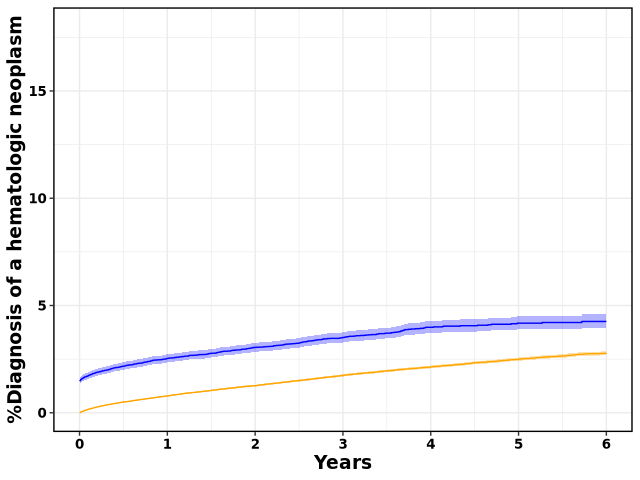
**

**References**

1. Elm E von, Altman DG, Egger M, Pocock SJ, Gøtzsche PC, Vandenbroucke JP. The Strengthening the Reporting of Observational Studies in Epidemiology (STROBE) statement: guidelines for reporting observational studies. *The Lancet*. 2007;370(9596):1453-1457. doi:10.1016/S0140-6736(07)61602-X

2. Halvorsen S, Ghanima W, Fride Tvete I, et al. A nationwide registry study to compare bleeding rates in patients with atrial fibrillation being prescribed oral anticoagulants. *Eur Heart J - Cardiovasc Pharmacother*. 2017;3(1):28-36. doi:10.1093/ehjcvp/pvw031

3. Hindricks G, Potpara T, Dagres N, et al. 2020 ESC Guidelines for the diagnosis and management of atrial fibrillation developed in collaboration with the European Association for Cardio-Thoracic Surgery (EACTS). *Eur Heart J*. 2021;42(5):373-498. doi:10.1093/eurheartj/ehaa612

4. Quan H, Li B, Couris CM, et al. Updating and validating the Charlson comorbidity index and score for risk adjustment in hospital discharge abstracts using data from 6 countries. *Am J Epidemiol*. 2011;173(6):676-682. doi:10.1093/aje/kwq433

5. Segal JB, Chang HY, Du Y, Walston JD, Carlson MC, Varadhan R. Development of a Claims-based Frailty Indicator Anchored to a Well-established Frailty Phenotype. *Med Care*. 2017;55(7):716. doi:10.1097/MLR.0000000000000729

6. ICD - Classification of Diseases, Functioning, and Disability. Published June 29, 2023. Accessed April 5, 2024. https://www.cdc.gov/nchs/icd/index.htm

7. Nomenclatuur van de geneeskundige verstrekkingen | RIZIV. Accessed April 5, 2024. https://www.riziv.fgov.be/nl/nomenclatuur

8. ATCDDD - Home. Accessed April 5, 2024. https://atcddd.fhi.no/
